# Identifying transcription start sites and active enhancer elements using BruUV-seq

**DOI:** 10.1038/srep17978

**Published:** 2015-12-11

**Authors:** Brian Magnuson, Artur Veloso, Killeen S. Kirkconnell, Leonardo Carmo de Andrade Lima, Michelle T. Paulsen, Emily A. Ljungman, Karan Bedi, Jayendra Prasad, Thomas E. Wilson, Mats Ljungman

**Affiliations:** 1 Department of Radiation Oncology, University of Michigan Comprehensive Cancer Center and Translational Oncology Program, University of Michigan Medical School, Ann Arbor, Michigan, USA; 2 Bioinformatics Program and Department of Computational Medicine and Bioinformatics, University of Michigan, Ann Arbor, Michigan, USA; 3 Department of Human Genetics, University of Michigan Medical School, Ann Arbor, Michigan, USA; 4 Department of Pathology, University of Michigan Medical School, Ann Arbor, Michigan, USA; 5 Department of Microbiology, Biomedical Sciences Institute, University of Sao Paulo, Brazil; 6 Department of Environmental Health Sciences, School of Public Health, University of Michigan, Ann Arbor, Michigan, USA

## Abstract

BruUV-seq utilizes UV light to introduce transcription-blocking DNA lesions randomly in the genome prior to bromouridine-labeling and deep sequencing of nascent RNA. By inhibiting transcription elongation, but not initiation, pre-treatment with UV light leads to a redistribution of transcription reads resulting in the enhancement of nascent RNA signal towards the 5′-end of genes promoting the identification of transcription start sites (TSSs). Furthermore, transcripts associated with arrested RNA polymerases are protected from 3′–5′ degradation and thus, unstable transcripts such as putative enhancer RNA (eRNA) are dramatically increased. Validation of BruUV-seq against GRO-cap that identifies capped run-on transcripts showed that most BruUV-seq peaks overlapped with GRO-cap signal over both TSSs and enhancer elements. Finally, BruUV-seq identified putative enhancer elements induced by tumor necrosis factor (TNF) treatment concomitant with expression of nearby TNF-induced genes. Taken together, BruUV-seq is a powerful new approach for identifying TSSs and active enhancer elements genome-wide in intact cells.

The principal steps of the transcriptional cycle are initiation, elongation and termination. Initiation occurs after recruitment of RNA polymerase II to promoter elements by transcription factors and by the aid of active enhancer elements^1^,^2^. Many genes employ multiple promoters that allow for differential RNA isoform expression, a fundamental component of tissue diversity and response to environmental changes^1^,^3^,^4^,^5^. A number of strategies exist for identifying potential transcription start sites (TSS) and enhancer elements on a genome-wide scale. One approach is ChIP-seq analysis of either transcription initiation complexes^6^ or specific histone modifications such as H3K4me1, H3K4me3 and H3K27ac^1^. ChIP-seq on its own, however, does not conclusively demonstrate promoter or enhancer usage but rather indicates the potential of a chromatin region or transcription initiation complex to be used as a TSS or enhancer. Another approach to identify TSS and enhancers genome-wide involves tagging of the 5′-ends of transcripts, examples of which include “cap analysis gene expression” (CAGE)^7^,^8^, “5′-end serial analysis of gene expression” (SAGE)^9^ and “gene identification signature” analysis (GIS) with “pair end tags” (PET)^10^. These techniques are very accurate in providing single-nucleotide resolution of TSSs, however, since these approaches rely on steady-state RNA they cannot account for alterations due to post-transcriptional processing. Furthermore, it has been reported that some recapping of degradation products of mature forms of RNA may occur in the cytoplasm and therefore some false-positive TSS and enhancer predictions may occur when using CAGE-based assays^11^.

RNA Pol II can generate RNA from enhancer elements, leading to the production of enhancer RNA (eRNA) whose function is unclear. eRNA is often transcribed bi-directionally but may not be polyadenylated and thus may be excluded from poly(A)RNA-based assays^12^. The chromatin surrounding enhancer elements is generally characterized by high H3K4me1 and H3K27ac modifications, binding of the acetyltransferase P300, and low levels of H3K4me3 modifications^13^. Genome-wide annotations based on specific histone modifications have allowed for the identification of thousands of putative enhancer elements^14^. However, not all putative enhancers generate eRNA. Those that do, often score higher in *in vitro* assays for enhancer activity, suggesting that production of eRNA is linked to functional activity^15^. RNA sequencing approaches using nascent RNA as a starting material, such as GRO-seq^16^ and bromouridine labeling and sequencing (Bru-seq)^17^, have been developed to study this nascent RNA pool. GRO-seq enables the detection of unstable RNA species generated from enhancer elements due to reduced RNA degradation in nuclei preparations^18^. Additionally, derivative techniques such as PRO-seq^19^ and GRO-cap^20^ can identify enhancers in a sensitive, high-resolution manner. Because the *in vitro* nuclear run-on assay activates paused RNA polymerases and promotes elongation, it is difficult to conclude that the measured levels of all eRNAs accurately reflect *in vivo* activity.

Here we present BruUV-seq, an approach that complements the Bru-seq technique by enhancing nascent RNA signal around promoters and enhancers genome-wide in intact cells. UVC light (254 nm) introduces predominantly cyclobutane pyrimidine dimers and 6–4 photoproducts in DNA that are distributed more or less randomly in the genome^21^. These lesions are strong transcriptional blocks, causing RNA polymerase II elongation complexes to stall^22^,^23^. In BruUV-seq, such lesions are introduced by UV irradiation prior to the metabolic labeling of nascent RNA with bromouridine (Bru), isolation of Bru-RNA, and deep sequencing (Fig. 1a). Although elongating RNA polymerases stall at UV-induced lesions within gene bodies, new initiation and transcription near active TSS and enhancer elements is expected to continue^22^,^23^. The net result is an increase in RNA read density at TSSs and at active enhancer elements. Furthermore, RNA species associated with stalled RNA polymerases are expected to be protected from 3′–5′ degradation by nuclear RNA exosomes as long as their 3′-ends are attached to the polymerases. Thus, transcription stalling by UV light enriches the transcription reads of unstable transcripts. BruUV-seq can thus readily identify TSSs and active enhancers as well as unstable transcripts genome-wide in intact cells, and assess changes in transcription levels both at promoter and enhancer regions following exposure to a stimulus or stress.

## Results

### UV light blocks elongation and redistributes RNA reads to TSSs

**Figure 1:**
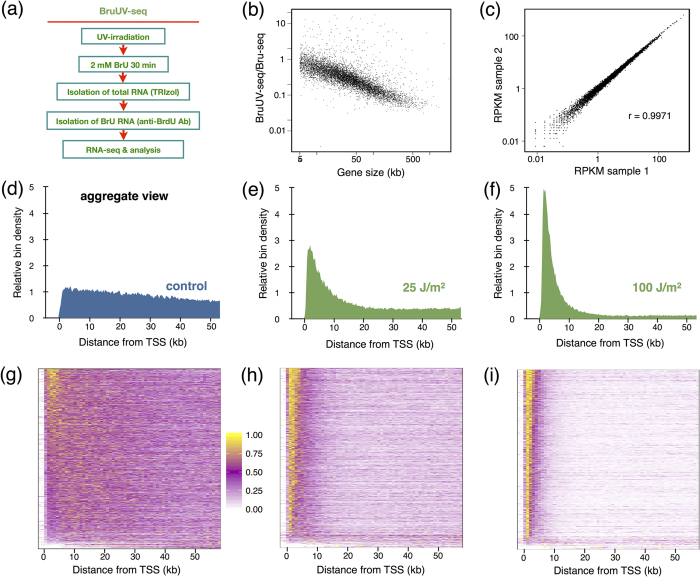
(**a**) Experimental outline of the BruUV-Seq technique. (**b**) Strong correlation between UV-mediated inhibition of transcription and gene size. The ratio of integrated transcription reads over whole genes following UV-irradiation in HF1 human fibroblasts with 20 J/m^2^ compared to mock-irradiated HF1 cells are shown on the Y-axis while gene size is shown on the X-axis. (**c**) Reproducibility of BruUV-seq. The RPKM values of the first 5 kb of genes expressed above 0.5 RPKM were compared between two biological experiments involving human HF1 fibroblasts in which cells had been UV-irradiated with 20 J/m^2^, Bru-labeled and sequenced in parallel. The Pearson’s correlation coefficient between these biological replicates was r = 0.9971. (**d**) BruUV-seq signal aggregated across genes from K562 cells that had been mock-irradiated, (**e**), irradiated with UV at 25 J/m^2^ or (**f**) 100 J/m^2^ Genes selected for analysis were at least 50 kb long and expressed more than 1 RPKM in the mock-irradiated sample (1225 genes). Genes were aligned by annotated TSS and median signal was calculated for 250 bp bins and normalized against the mock-irradiated sample. (**g**–**i**) Heat maps representing genes (selected as in *d*) on the vertical axis, and 1 kb bins from −2 kb to + 60 kb on the horizontal axis for (**g**) mock-irradiated, (**h**) 25 J/m^2^ UV, and (**i**) 100 J/m^2^ UV. For each gene, bin values were scaled to a 0–1 range and sorted by the sum of scaled values in the first 5 kb following the TSS.

It is predicted that inhibition of gene expression by UV light is proportional to both the dose of UV light and the size of the gene. Indeed, introducing random, transcription-blocking lesions by UV light has been used to determine genomic sizes of individual genes^24^. To test what effect gene size has on the inactivation of nascent RNA synthesis on a genome-wide scale, we mock-irradiated or irradiated normal human fibroblasts (HF1 cells) with 20 J/m^2^ of UVC light (254 nm) and immediately labeled nascent RNA with 2 mM bromouridine (Bru) for 30 min. UVC-irradiation has been determined to induce on average 1 transcription-blocking lesion (CPD or 6-4PP) per 100 kb in the transcribed strand per each J/m^2^ 25. Therefore, we expect 1 lesion/5 kb with a 20 J/m^2^ UVC dose. Bru-labeled RNA was isolated and subjected to deep sequencing (see Fig. 1a, Methods, and ref. 25). A strong negative correlation was observed between the ratio of UV-irradiated to mock-irradiated RPKM values and gene size (Fig. 1b). Thus, the larger the gene, the less overall nascent RNA synthesis signal obtained following UV-irradiation, in agreement with our recent findings in human fibroblasts^26^. We also found that, similar to Bru-seq^17^, BruUV-seq was reproducible when comparing two biological replicates (Pearson’s r = 0.9971, Fig. 1c).

We next examined the effect of UV-irradiation on the distribution of RNA reads within genes in human K562 cells. Genes of at least 60 kb in length and expressed at least 1 RPKM in the Bru-seq (mock-irradiated) sample were aligned by their annotated TSSs and divided into 250 bp bins. An aggregate view representing the median RPKM per bin across 1225 genes was produced (Fig. 1d–f). Nascent Bru-seq data exhibited a relatively even distribution of signal from the TSS into the gene (Fig. 1d). Following exposure to 25 J/m^2^ (Fig. 1e) or 100 J/m^2^ (Fig. 1f) of UVC radiation, the read distribution shifted markedly towards the TSS in a dose-dependent manner. Redistribution of reads on a per-gene basis is represented by heat maps of 1 kb bins aligned by annotated TSSs (Fig. 1g–i). The nearly uniform redistribution of reads across all expressed genes indicates that this effect is predominantly due to the UV-induced elongation blockage. The increase in reads captured near TSSs are relative and do not necessarily represent UV-induced induction of transcription but rather reveal TSS utilization based on the redistribution of nascent RNA reads following UV-irradiation. However, UV light trigger a DNA damage response that during the 30 min labeling period results in the activation and repression of transcription initiation of target genes so the magnitude of the UV-induced peak may differ between certain genes.

### Identification of active TSSs using BruUV-seq

**Figure 2:**
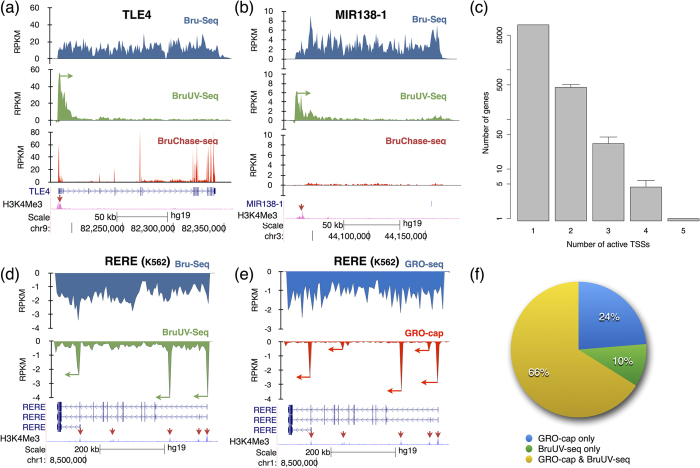
BruUV-seq identifies TSSs genome-wide. (**a**) BruUV-seq, using 20 J/m^2^ UVC, verifies that the *TLEA4* gene in HF1 cells is transcribed from a single TSS that aligns with H3K4me3 marks (red arrow). (**b**) The primary transcript of MIR138–1 is initiating transcription from a single TSS about 80 kb upstream of the *MIR138-1* sequence. (**c**) Genome-wide assessment of the number of TSSs per gene detected with BruUV-seq using HF1 cells and shows that many genes have multiple TSSs. Data is taken from three independent biological experiments with bars showing the standard deviation. (**d**) BruUV-seq (100 J/m^2^ UVC) identifies three distinct TSSs in the *RERE* gene in K562 cells. (**e**) GRO-cap identifies three strong and two minor TSSs for *RERE* in K562 cells. (**f**) Annotated TSSs identified as active in BruUV-seq (100 J/m^2^ UVC) and GRO-cap. 5720 TSSs represented for genes that expressed at least 1 RPKM in the K562 Bru-seq sample. GRO-cap data was from GEO (GSE60456)^18^. The H3K4me3 ChIP-seq data are from ENCODE for normal human lung fibroblasts (NHLF) (**a,b**) and K562 cells (**d**,**e**).

To explore the behavior of individual genes, we performed Bru-seq to obtain genome-wide transcription rates, BruUV-seq to assess TSS usage, and our previously described BruChase-seq technique to assess splicing and turnover after a 6-hour uridine chase^17^,^27^. We also compared nascent RNA expression with histone modification tracks generated by ENCODE (UCSC genome browser) for K562 cells or normal human lung fibroblasts (NHLF), the best cell type match for HF1. UV-induced read-redistribution is demonstrated by the*TLE4 gene*, where BruUV-seq produced one peak at the proximal boundary of the Bru-seq signal span that corresponds to the single putative TSS as predicted by its single H3K4me3 peak (Fig. 2a). While the Bru-seq data verifies that this gene is transcribed in HF1 cells, the BruChase-seq data demonstrates that this gene produces a typical, spliced mRNA that is relatively stable. Together, Bru-seq, BruUV-seq and BruChase-seq provide a comprehensive picture of the transcriptional and post-transcriptional regulation of *TLE4* and other genes.

Combining BruUV-seq with Bru-seq and BruChase-seq allowed us to better describe poorly-annotated transcription units. For example, the primary transcripts of miRNAs are not well-annotated because they are rapidly processed into mature 22-mers while the rest of the primary transcript is degraded. Bru-seq revealed a long nascent transcript emanating some 80 kb upstream of the annotated mature *MIR138-1* DNA sequence in HF1 cells (Fig. 2b). A single BruUV-seq peak located the same distance upstream suggests that this is the TSS of the *MIR138-1* primary transcription unit. Indeed, this conclusion is supported by an H3K4me3 peak found at the same location in NHLF cells. BruChase-seq confirmed that the primary miRNA transcript was very unstable since very few reads for this transcript were found after the 6-hour chase.

Differential TSS usage is a major contributor to transcript diversity in cells. To determine the extent of TSS usage genome-wide in HF1 cells, BruUV-peaks at annotated TSSs were measured. Gene isoforms with identical TSSs were merged in the annotation. Due to the observed broad width of BruUV-seq signal near the TSS (often ~5 kb, see Fig. 1f,i), gene isoforms with non-identical TSSs within 1 kb of each other were merged since they could not be detected individually and the most upstream TSS was used. Active TSSs were identified using a Hidden Markov Model, which identifies the characteristic BruUV-seq peaks (see Methods). This method was capable of resolving peaks 1 kb apart or more. We found that the majority of expressed genes (~95%) in human fibroblasts had only one active TSS, while a few genes utilized up to 5 distinct TSSs (Fig. 2c). The *RERE* gene is an example of a gene with multiple putative TSSs as suggested by multiple H3K4me3 peaks and BruUV-seq peaks were observed on three of these putative TSSs (Fig. 2d). Additional genes utilizing multiple TSSs are shown in Supplementary Fig. 1.

Recent methods for high-resolution TSS identification have been described, so we next compared peaks generated by BruUV-seq at putative TSSs with data for these other techniques. Both CAGE and GRO-cap are techniques used to identify TSSs by capturing RNA molecules via their 5′-CAP. Because CAGE analyzes RNA from the steady-state pool rather than from the nascent pool, direct comparison with BruUV-seq is not very meaningful. However, a qualitative comparison is provided in Supplementary Fig. 2. Despite the high sensitivity of the technique, CAGE signal is sometimes absent at a TSS where a BruUV-seq peak is evident, either due to differences between the two independent studies, or because of fast turnover rates or lack of 5′-end capping of some RNA species. GRO-cap (RNA sequencing following a limited nuclear run-on reaction and 5′ CAP capture^18^) is more comparable to BruUV-seq because of its nascent RNA scope. GRO-seq and GRO-cap public data for the RERE gene from K562 cells (Fig. 2e) mimic the data for Bru-seq and BruUV-seq. Importantly, all three peaks obtained with BruUV-seq correspond to peaks obtained with GRO-cap. In addition, GRO-cap identified two smaller peaks that were not detected by BruUV-seq. Due to the concentration of the GRO-cap reads to the capped sequences, a much higher depth can be obtained at these sequences.

In order to compare GRO-cap data with BruUV-seq data genome-wide, we carried out the active TSS identification analysis (with some parameter adjustments) to publicly available GRO-cap data from K562 cells^18^. From the union set of BruUV-seq and GRO-cap results (12,503 TSSs) we selected those where the respective genes were expressed above 1 RPKM in the K562 Bru-seq sample for comparison (5702 TSSs). Sixty-six percent of these active TSSs were identified by both BruUV-seq and GRO-cap (Fig. 2f). Furthermore, 10% and 24% of the remaining TSSs were identified by either BruUV-seq or GRO-cap, respectively.

### Are some gene clusters transcribed as operons in human cells?

Gene clusters transcribed in the same orientation sometimes share a common promoter and are transcribed as a “neighborhood” or operon. This polycistronic arrangement is common in bacteria and in some eukaryotes such as *C. elegans* and *D. melanogaster*^28^,^29^ but evidence for such operons in human cells is scarce. Polycistronic miRNAs have been identified in human cells as well as transcription from rRNA clusters and from the mitochondrial genome. Furthermore, polycistronic snoRNAs have been reported in eukaryotes, including *D. melanogaster*, but are thought to be absent in humans^30^. To explore whether some gene clusters in human cells are co-expressed from a single TSS, Bru-seq and BruUV-seq data from human fibroblasts were compared. For example, a gene cluster coding for zinc finger proteins on chromosome 12 was shown by Bru-seq to be transcribed at similar levels and in the same orientation (a potential operon), yet BruUV-seq revealed that each of the genes in this cluster utilized their own TSS (Supplementary Fig. 3a). In contrast, a region on chromosome 14 encoding a large set of snoRNAs and miRNAs appeared to be transcribed from a single common TSS (Supplementary Fig. 3b). For protein-coding genes we found little evidence for polycistronic transcription but *TTTY15* and *USP9Y* on the Y chromosome appeared to fit this paradigm (Supplementary Fig. 3c, bottom panel). Both genes are synthesized on the same strand and spliced, as demonstrated by the exonic peaks in the BruChase-seq trace Bru-seq, suggesting a single long transcription unit spanning both genes (Supplementary Fig. 3c, top panel) with only one BruUV-seq peak present at the 5′ end of the upstream gene (Supplementary Fig. 3c, middle panel). The neighboring TTTY15 and USP9Y genes have been reported to operate as fusion genes in prostate cancer^31^ and thus it is possible that this finding in K562 cells is due to a gene fusion. Taken together, these data illustrate that the BruUV-seq approach can identify the site of transcription initiation, whether multiple promoters are used for a given gene and, in combination with Bru-seq, whether genes are organized into neighborhoods utilizing a common promoter. While BruUV-seq revealed evidence for polycistronic organization of miRNA and snoRNA gene clusters, no evidence was found that such gene organization is common for protein-coding genes.

### Use of BruUV-seq to validate gene fusions

Gene fusions are the products of aberrant recombination of normally separate genes. Many identified gene fusions are oncogenes known to cause or contribute to cancer^32^,^33^. Chronic myelogenous leukemia (CML) is caused by a chromosomal translocation between chromosomes 9 and 22 that fuses the 5′ portion of the *BCR* gene to the 3′ portion of the *ABL1* gene^34^. In K562 cells, a CML-derived cell line known to harbor *BCR-ABL*, Bru-seq revealed high *BCR* expression throughout most of the 5′-part of the gene with a drastic drop in RNA reads at the known translocation site near the 3′-end of the gene (Supplementary Fig. 4a, left panel, red arrow). The *ABL1* gene, on the other hand, exhibited low expression at its 5′-end, which increased dramatically about 10 kb into the gene (Supplementary Fig. 4a, right panel, red arrow). In the absence of prior knowledge of a gene fusion at this locus, the abrupt increase in signal from the *ABL1* gene could be interpreted as initiation from a strong, un-annotated promoter located in the first intron. However, BruUV-seq showed no peak to support this notion (Supplementary Fig. 4b, right panel, red arrow), but did identify the strong active *BCR* TSS known to drive *BCR*-*ABL* expression (Supplementary Fig. 4b, right panel, red arrow). Thus, BruUV-seq can be used to clarify and validate the existence of gene fusions in cells and could potentially be used to predict the location of patient-specific translocation junctions.

### Using BruUV-seq to assess induced initiation of transcription

**Figure 3:**
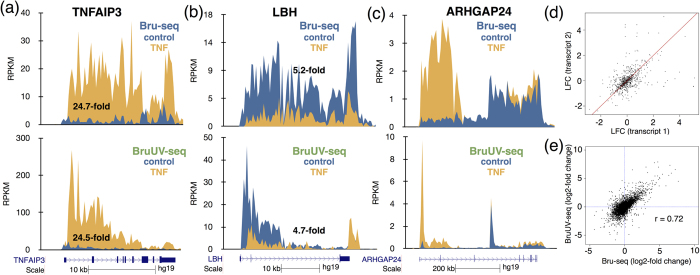
BruUV-seq can be used to predict Bru-seq data for gene induction. (**a**) *Top:* The *TNFAIP3* gene is induced 24.7-fold following treatment with TNF in HF1 cells as measured with Bru-seq^17^. *Bottom:* Comparing the read density over the first 5 kb of the *TNFAIP3* gene of control and TNF-treated cells, we found that BruUV-seq (20 J/m^2^ UVC) data yield a 24.5-fold enrichment by TNF, which is similar to the 24.7-fold induction measured with Bru-seq at top. (**b**) *Top:* Transcription from the *LBH* gene is reduced 5.2-fold measured with Bru-seq and 4.7-fold measured over the first 5 kb using BruUV-seq at bottom. (**c**) Isoform-specific induction of the *ARHGAP24* gene shown with Bru-seq data on top is confirmed with BruUV-seq at bottom. (**d**) Pairwise comparison of the TNF-induced log-fold change in gene expression of transcripts in genes with multiple active TSS. Points distant from the identity line (red line) indicate genes with TSS-specific response to TNF. (**e**) Correlation between TNF-induced changes in gene expression using the full gene lengths in Bru-seq (x-axis) and the first 5 kb of the genes in BruUV-seq (y-axis). The Pearson’s r = 0.72.

To assess whether BruUV-seq data could be used to infer expression levels over entire genes, we compared the BruUV-seq signal from the first 5 kb of genes with the relative Bru-seq signal from the entire gene and found a strong correlation (Supplementary Figs 5–7). We next explored whether BruUV-seq could accurately capture changes in gene expression following TNF-mediated induction of the acute inflammatory response^17^. *TNFAIP3* is a gene that was found to be induced by about 25-fold by TNF as assessed by Bru-seq (Fig. 3a). Using BruUV-seq to assess the fold difference in transcription reads over the first 5 kb between control and TNF treated cells, we observed a 24-fold difference. The *LBH* gene was found to be down-regulated 5.2-fold by TNF as measured by Bru-seq and 4.7-fold when measured over the first 5 kb using BruUV-seq (Fig. 3b). The *ARHGAP24* gene showed an isoform-specific TNF-induction, which is clear in the Bru-seq data (Fig. 3c, top panel). We next performed pairwise comparison of the TNF-induced log-fold change in gene expression of transcripts in genes with multiple active TSS. Points distant from the identity line (red line) indicate genes with TSS-specific response to TNF (Fig. 3d). Genome-wide comparison of TNF-mediated changes in transcription between Bru-seq and BruUV-seq suggests a moderately high correlation (Pearson’s r = 0.72) (Fig. 3e). Due to overlapping of isoforms, however, the accurate assignment of reads to specific isoforms is not a trivial problem. When TSSs are well separated, BruUV-seq was actually able to reveal both TSS usage (and therefore isoform usage) as well as the expression levels of these isoforms. Thus, measuring changes in transcription of the first 5 kb of genes using BruUV-seq can be used as a reasonable surrogate for Bru-seq measuring changes across whole genes or transcripts. This is particularly important when measuring transcription induction of large genes from nascent RNA sequencing data, as more time is required for the wave of induced transcription to reach the 3′-end of the gene and thus, measuring transcription reads from the whole gene will underestimate the treatment-induced induction of these large genes.

### UV light increases read density at putative enhancer elements

**Figure 4:**
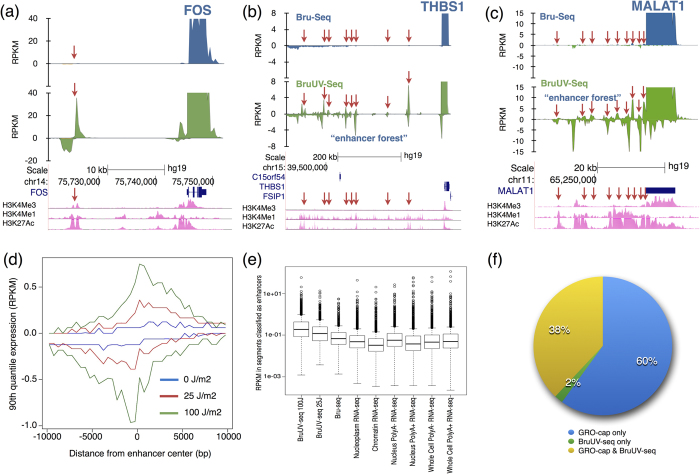
Use of BruUV-seq to identify active enhancer elements genome-wide. (**a**) Bru-seq (blue) and BruUV-seq (20 J/m^2^ UVC) (green) data for the *FOS* gene and upstream enhancer element (red arrow) in HF1 cells. Bru-seq and BruUV-seq data for an “enhancer forest” region (red arrows) upstream of the highly expressed *THBS1* (**b**) and *MALAT1* (**c**) genes in HF1 cells. (**d**) An aggregate view of the reads surrounding 526 intergenic enhancer regions as defined by the ENCODE project’s combined genome segmentation annotation in K562 cells. It can be seen that the aggregate signal of eRNA was bidirectional and the enhancement of reads was proportional to the UV dose. (**e**) Boxplot indicating distribution of expression values (RPKM) observed in intergenic ENCODE-defined enhancer regions in K562 cells. Data includes BruUV-seq, Bru-seq and ENCODE’s cellular fractionation long RNA-seq samples. (**f**) Comparison of intergenic BruUV-seq (100 J/m^2^ UVC) and GRO-cap peaks overlapping ENCODE enhancers. GRO-cap data was from GEO (GSE60456)^18^.

**Figure 5:**
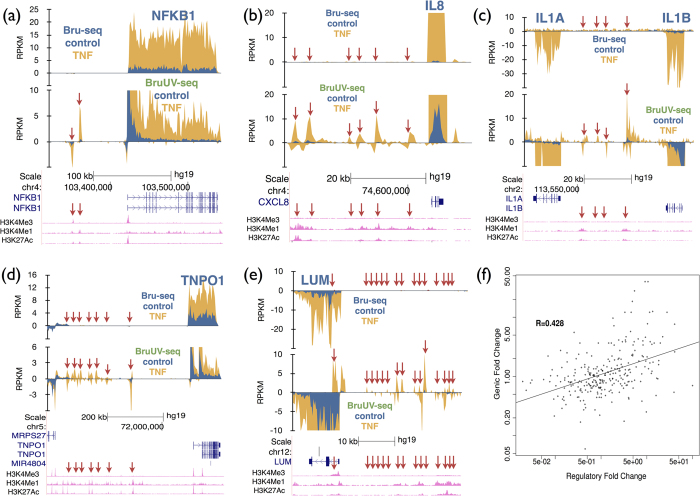
Use of BruUV-seq to identify enhancer elements induced by TNF. Comparison of Bru-seq (top) and BruUV-seq (20 J/m^2^ UVC) (bottom) for the (**a**) *NFKB1*, (**b**) *IL8,* (**c**) *IL1A and IL1B,* (**d**) *TNPO1 and* (**e**) *LUM* genes and upstream regions in HF1 cells before and after treatment with TNF for 1 hour. Enhancer elements activated by TNF are shown with red arrows. (**f**) Correlation between TNF-induced changes in eRNA and mRNA expression in human fibroblasts. Enhancers were identified using a Hidden Markov Model based on the BruUV-seq and Bru-seq expression profiles and were assigned to their nearest genes. The histone modification tracks are from ENCODE for normal human lung fibroblasts (NHLF).

Certain putative enhancer elements have been shown to generate RNA (eRNA)^12^. It is not well understood how the eRNA is produced and what function it may have in regulating genes. The fact that the eRNA is capped and the genomic similarities between enhancers and certain groups of promoters^15^ suggest that active enhancers may behave similar to promoters. We therefore predicted an increase in BruUV-seq signal around enhancers producing eRNA. Indeed, RNA reads generated by BruUV-seq were enriched in narrow peaks in intergenic regions corresponding to known enhancer elements. An example of such a peak was located upstream of the *FOS* gene in a region of a well-known enhancer element (Fig. 4a). Interestingly, certain genomic regions had a high concentration of BruUV-seq peaks coinciding with high H3K4me1 and H3K27ac and low H3K4me3 marks. Two such regions are indicated in Fig. 5b,c, which we refer to as “enhancer forests”, located upstream from *THBS1* and the non-coding *MALAT1* gene. These “enhancer forests” are likely describing super enhancers or stretch enhancers^35^,^36^.

In addition to a redistribution of reads from the bodies of genes to TSSs, we hypothesized that eRNA signal may be enhanced following UV-irradiation due to stabilization when associated with RNA polymerases stalled at UV-induced lesions. The basis for this is that the 3′-end of the transcripts should be protected against the 3′–5′ RNase activity of the nuclear RNA exosome^37^ while associated RNA polymerase. Indeed, Bru-labeled RNA was turned over more slowly if the cells were UV-irradiated and even more so with continuous treatment with the transcription elongation inhibitor actinomycin D after the Bru-labeling (Supplementary Fig. 8). Furthermore, UV light was recently shown to inhibit RNA exosome activity via p38MAPK/MK2-mediated phosphorylation of RBM738, 39. Thus, the enhancement of nascent RNA reads over enhancer elements with BruUV-seq is likely due to a combination of 3’-end protection by RNA polymerases stalled at UV lesions and stress-activated phosphorylation leading to inhibition of RNA exosome function.

In order to determine whether the intergenic BruUV-seq peaks observed coincided with enhancer elements, we compared our data to enhancers predicted by the combined genome segmentation annotation from ENCODE^14^. First, segments of enhanced BruUV-seq signal (UVE) from K562 cells were identified using a hidden Markov model-based approach (see Methods). Intergenic UVE and ENCODE enhancer segments were defined as those outside all active transcription units identified in the nascent RNA data (non-irradiated K562 Bru-seq). Genome segmentation into transcription units was carried out as previously described^17^,^27^. Then the signal was calculated for regions adjacent to the centers of ENCODE-classified enhancer segments that overlap the BruUV-seq segments. An aggregate view of such intergenic enhancer regions demonstrated that the signal was bidirectional, which is consistent with other reports describing eRNA (Fig. 4d)^12^. Enrichment of signal in intergenic enhancers was greater in BruUV-seq than in Bru-seq, and greater at a higher UV dose (Fig. 4d). We found that BruUV-seq signal over enhancers was greater than ENCODE K562 RNA-seq data, regardless of capturing poly(A)-plus, poly(A)-minus, or subcellular fractions of RNA, indicating its general sensitivity (Fig. 4e).

Because the nuclear run-on assay for GRO-seq and GRO-cap is performed *in vitro* and presumably in the absence of RNA-degrading enzymes, it is expected that unstable RNA species, such as eRNA, will be detected. Indeed, when we compare enhancer signal from BruUV-seq to previously published GRO-seq data from K562 cells, GRO-seq signal was similar in shape, but overall greater in magnitude (Supplementary Fig. 9a). Additionally, we detected a significant number of segments overlapping enhancers in the GRO-seq data that were not detected in BruUV-seq data (data not shown). We predicted that the same trend would apply to promoter upstream transcripts (PROMPTs) (another labile species of RNA) and indeed the GRO-seq divergent signal just upstream of TSSs in expressed genes is greater than that obtained using BruUV-seq (Supplementary Fig. 9b). GRO-seq data exhibit a known enrichment near the TSS, due to a concentration of initiated, but paused polymerases being released during the run-on assay. We found that BruUV-seq exhibits greater enrichment near the TSS than GRO-seq, as the BruUV-seq signal substantially drops off ~5 kb into gene bodies, whereas GRO-seq captures signal throughout the gene body and in this way appears similar to Bru-seq signal (Supplementary Fig. 9b).

GRO-cap is a method expected to specifically enrich for nascent, 5′ end-capped RNA including eRNA^18^. We reasoned that we could utilize our UV peak identification analysis on GRO-cap data (since it is similarly enhanced over the Bru-seq nascent RNA signal), and indeed it is highly enriched in global ENCODE-defined enhancer regions. We compared these GRO-cap regions to BruUV-seq regions and found that 38% of putative enhancer elements genome-wide had signal in both BruUV-seq and GRO-cap while 60% were identified by GRO-cap only and 2% by BruUV-seq only. Thus, the great majority of BruUV-seq peaks in the putative enhancer elements were corresponding to GRO-cap signals. However, a significant portion of GRO-cap-identified enhancer regions were missed by BruUV-seq. Since GRO-cap genome coverage is more focused than BruUV-seq, we expect it to be much more sensitive at a similar sequencing depth (for reference, we mapped 25,524,149 reads from the GRO-cap sample and 32,936,591 reads from the BruUV-seq (100 J/m^2^) sample). Thus, some of these GRO-cap-only enhancer segments may be explained by its higher sensitivity of these elements compared to BruUV-seq. Indeed, when we compared the intensity of the GRO-cap signal between the enhancers that are detected by both GRO-cap and BruUV-seq with enhancers detected by GRO-cap alone we found that high intensity GRO-cap signal was overrepresented in the group of enhancers detected by both methods (Supplementary Fig. 10). Some enhancer regions detected by GRO-cap but not BruUV-seq may represent run-on elongation of RNA polymerases that were arrested *in vivo*. Taken together, these data suggest that BruUV-seq identifies many, but not all, enhancer elements that are identified by GRO-seq and GRO-cap, which may reflect differences in sensitivity and differences between the *in vivo* vs. *in vitro* labeling steps of these two techniques.

### Changes in gene expression are accompanied by changes in eRNA production

It has been shown that the level of eRNA expression positively correlates with the expression level of their closest genes^12^. Here we tested whether such correlation could be detected using BruUV-seq in human fibroblasts exposed to TNF to induce the acute inflammatory response. With Bru-seq we observed a sharp TNF-mediated induction of *NFKB1* gene expression (Fig. 5a)^17^. Using BruUV-seq we observed two strong peaks about 40–50 kb upstream of the TSS for the *NFKB1* gene that aligned with the enhancer marks H3K4me1 and H3K27ac and the intensity of these two peaks increased dramatically following TNF treatment. Dramatic induction of UV-peaks was also seen nearby of highly TNF induced gene such as IL8, IL1A, IL1B, TNPO1 and LUM (Fig. 5b–e).

Based on such examples, we reasoned that the ratio between BruUV-seq and Bru-seq signal before and after a treatment could be used to identify enhancers that become activated following treatment. First, we searched for BruUV-seq peaks in and around genes that were induced 1 hour following TNF treatment. Out of the top 99 genes induced by TNF, 15 genes had more than 5 associated putative TNF-activated enhancer elements (i.e. BruUV-seq peaks induced by TNF but not detected in Bru-seq data). Furthermore, 50 genes had 1–5 induced enhancer elements while 34 genes appeared to be induced by TNF in an enhancer-independent way (Supplementary Table I).

We next used a hidden Markov model to identify intergenic regions with enhanced BruUV-seq signal (UVE regions). The TNF-induced change in RNA production in these UVE regions was then compared to the TNF-induced change in pre-mRNA expression of their nearest gene. A positive correlation was observed (r = 0.428), suggesting that changes in gene expression and nearby, putative eRNA production are correlated genome-wide (Fig. 5f). Not all enhancers are interacting with their nearest neighbor so our estimate of the correlation between induction of eRNA and induction of its cognate gene is an underestimate. Taken together, the BruUV-seq technique presented here helps identify both TSSs and enhancer utilization genome-wide and can assess changes in TSS and enhancer activity when homeostasis is altered.

## Discussion

Here we present BruUV-seq as a companion technique to Bru-seq, BruChase-seq^17^,^27^ and BruDRB-seq^40^ where cells are UV-irradiated prior to metabolic labeling of nascent RNA (Fig. 1a). Due to inhibition of transcription elongation randomly throughout the genome, UV pretreatment redistributes the bromouridine labeling of nascent RNA toward the beginning of transcription units. This is due to the combined action of blocked transcription elongation in the bodies of genes without inhibiting transcription initiation. In addition, ordinarily unstable RNAs, such as eRNA, are protected from degradation when persistently bound to stalled RNA polymerases and through UV-induced inactivation of RNA exosome components^38^,^39^, leading to the markedly increased yields of reads corresponding to eRNAs and PROMPTS. These effects of UV light form the basis of the BruUV-seq approach and allow for the identification of active TSSs and enhancer elements genome-wide.

Combined analysis of Bru-seq and BruUV-seq data provides novel insight into nascent transcription and context-dependent gene function. Transcription inhibition by UV light is proportional to gene size, confirming the findings by Sauerbier and colleagues when estimating genomic sizes of individual genes^24^ (Fig. 1b). It has been proposed that UV light may cause inhibition of transcription initiation^41^ but our data presented here and previously^26^, suggest that transcription elongation is the primary target of UV-mediated inactivation of transcription. We were able to identify thousands of active TSSs genome-wide, with hundreds of genes presenting more than one active TSS, most likely giving rise to distinct transcript isoforms (Fig. 2). We found examples of gene clusters driven by individual promoters (Supplementary Fig. 3a) as well as gene clusters driven by a single TSS (Supplementary Fig. 3b,c). Furthermore, we detected multi-TSS containing genes where the different TSSs responded differently to a specific stimulus (e.g. Fig. 3c). In the K562 cell line, BruUV-seq confirmed the presence of the *BCR-ABL* gene fusion by showing that this fusion gene initiated from a single TSS in the *BCR* gene (Supplementary Fig. 4).

In addition to identifying TSS usage, we found that BruUV-seq data from the first 5 kb of genes could be used to infer transcription levels of the entire gene. This application of BruUV-seq proved especially important for very large genes or when genes harbor multiple active TSS, since each TSS can be identified and analyzed independently immediately after a treatment-induced change in transcription. BruUV-seq also enabled us to observe treatment-induced changes in eRNA production (Fig. 5). Many of the most highly TNF-induced genes showed activation of multiple BruUV-seq peaks or “enhancer forests” that we believe represent super enhancers or stretch enhancers (Fig. 5, Supplementary Table I). In addition, we identified TNF-induced genes that appeared to be induced in and enhancer-independent manner. The positive correlation observed between treatment-induced changes in eRNA and pre-mRNA is important since it suggests that most of these *in silico*-identified sites are in fact regulators of the selected genes.

## Conclusions

Much effort has been given to map enhancer elements by identifying specific histone marks and transcription factor binding sites using ChIP-seq and connecting these peaks with activated gene expression using RNA-seq. Tools such as DNase hypersensitivity sites^42^, ChIA-PET^43^, GRO-seq^18^, GRO-cap^18^ and CAGE^15^ have also been utilized to interrogate relationships between regulatory regions and gene transcription. We believe that the abilities of BruUV-seq to simultaneously assess nascent gene transcription and inform TSS and enhancer usage makes it orthogonal and complementary to current techniques for exploring the mechanisms of gene regulation genome-wide. Furthermore, when BruUV-seq is combined with Bru-seq, BruChase-seq and BruDRB-seq, which are based on the same labeling and analysis platform, many novel aspects of transcriptional and post-transcriptional regulation of gene expression can be interrogated in the same experiment.

## Methods

### Cell culturing

Human diploid foreskin fibroblasts HF1 (a gift from Mary Davis, University of Michigan) expressing hTERT^17^,^27^,^40^,^44^,^45^ were grown as monolayers in MEM supplied with 10% fetal bovine serum and antibiotic-antimycotic solution (Invitrogen). K562 cells were grown in suspension in IMDM with 10% FBS.

### UV-irradiation and bromouridine labeling of cells

The media of adherent cells grown on 100 mm plates was removed and 100 μl of PBS was added to keep the cells from drying out during the UV-irradiation. Suspension cells were gently pelleted and suspended in 1 ml of PBS and placed in a 100 mm plates for UV-irradiation at room temperature. Cells were irradiated in 100 mm plates without the lid on with different doses of 254 nm UVC light. The irradiation source (Philips, New York, NY) generated UVC light with a dose rate of 1 J/m^2^/s as measured with a UVX radiometer (UVP, Inc. Upland CA). Immediately following UVC irradiation, the cells were supplied with conditioned media containing 2 mM bromouridine (Bru) (Aldrich) and they were incubated for 30 minutes to label nascent RNA. Isolation of Bru-containing RNA, cDNA library preparations and deep sequencing were performed as previously described^17^,^27^.

### Read mapping and gene annotations

Sequenced reads (strand-specific, single-end, 52 bp, and untrimmed) were initially mapped to human ribosomal DNA complete repeating unit (U13369.1) using Bowtie v0.12.8^46^. Reads that remained unaligned were mapped to the human genome build hg19/GRCh37^17^,^27^ using TopHat v.1.4.1^47^. The NCBI RefSeq^48^ annotated isoforms were merged to create a simplified annotation with one entry for each gene, as previously described^17^,^27^. This simplified annotation was used for all analyses in this manuscript except for TSS usage identification. For the TSS analysis (described below), the Ensembl gene annotation (release 69)^49^ was used and modified such that isoforms with identical TSSs were merged and isoforms with different TSSs within 1 kb of each other were also merged, assigning the most upstream TSS to the modified annotation.

### Identification of active TSSs

A two state Hidden Markov Model (HMM), similar to the one described by Veloso *et al.*^40^, was used to determine if a peak in the BruUV-seq signal occurred within 500 bp of an annotated TSS. This model attempted to recognize a state prior to the TSS (state 1), characterized by low signal, and a state after the TSS (state 2), characterized by a large increase in signal. BruUV-seq signal was measured in 250 bp bins spanning the genomic region 5 kb upstream to 5 kb downstream of a given TSS. This data was first quantile-normalized followed by a z-score Gamma-equivalent normalization. The resulting output was used as the observed output of the bins for the purposes of the HMM. The signal from two regions near the TSS (5–2 kb upstream; 2–5 kb downstream) was used to determine the emission probabilities of states 1 and 2, consecutively, with the only possible transition being from state 1 to 2 and its transition probability set to 0.00001. The model was fit to the data and the Viterbi algorithm was used to determine the most likely state of each bin using the R package msm^50^,^51^. A modified Ensembl annotation was used for TSS identification (see above) and a given TSS was considered ”active” if a transition from state 1 to state 2 occurred within 500 bp of the annotated TSS. The same method and annotation was used to identify TSSs in public GRO-cap data, except the analysis region was narrowed to 500 bp upstream/downstream and divided into 50 bp bins, parameters better suited to the narrow peaks of GRO-cap. The training regions were also adjusted (−500 bp to −100 bp and 0 bp to 500 bp).

### Variance comparisons in Bru-seq and BruUV-seq (related to Supp. Fig. 7)

The mapped reads for different Bru-seq and BruUV-seq experiments were sampled without replacement to simulate libraries with progressively fewer numbers of mapped reads. For each sequencing depth (e.g. 2 M, 5 M, 10 M, 15 M, 20 M, 25 M, 30 M), 10 individual simulations were created by randomly choosing the desired number of reads from the previously mapped reads. Genes determined to have an active TSS according to the original data were used in this analysis. Their expression signal (RPKM) was measured for the simulated Bru-seq and BruUV-seq libraries. For Bru-seq, the whole annotated gene was used to determine the expression level, while only the first 5 kb downstream of the TSSs were used in the BruUV-seq analyses. The coefficient of variation (mean divided by standard deviation) in expression of each gene from the 10 simulations was calculated for each sequencing depth in each sample.

### Determining eRNA expression

Two approaches were taken to determine genomic regions that were likely to represent enhancers. The first approach was to measure signal at enhancer segments (class E) as annotated by ENCODE’s combined genome segmentation for K562 cells^14^. In the second approach, we carried out *de novo* discovery of regions with BruUV-seq signal enhancement (UVE; see Identification of UV enhancement peaks). eRNA was distinguished from genic RNA by including only intergenic UVE sites, defined as regions that do not overlap genes or their transcription units. Transcription units were determined in K562 cells by Bru-seq as previously described^17^. Signal in UVE regions was measured in RPKM. The same methods were also applied to previously published GRO-seq and GRO-cap data in order to compare with BruUV-seq.

### Identification of UV enhancement peaks

Relative to Bru-seq, BruUV-seq signal is enhanced just downstream of TSSs followed by a decrease at more distal gene positions (e.g. Fig. 2a). Based on this assumption, we developed a hidden Markov model with two states: UV enhanced (UVE) and UV repressed (UVR). Read counts for paired Bru-seq and BruUV-seq samples were first independently aggregated into 250 bp bins and subjected to wavelet smoothing. Paired data were then normalized to a common scale based on total sample read counts and the fraction of UV reads (f_UV_) determined for each bin, where each read was considered to be a Bernoulli trial with a sample-pair-dependent probability of being from the BruUV-seq sample. Bins in annotated genes with a Bru-Seq gene RPKM of at least 0.25 that were more than 20 Kb downstream of the TSS were then used to determine the cumulative f_UV_ in the presumptive UVR portion of each gene. The mean and variance of these f_UV_ values were used to calculate the α and β shape parameters of the binomial distribution with over dispersion of f_UV_, i.e. the beta binomial distribution, for the UVR state. Similar shape parameters were obtained for the UVE state by reflecting the UVR beta distribution such that bins with a high probability of being UVR had a low probability of being UVE. Emissions probabilities for the UVR and UVE states were then calculated for each bin using these shape parameters and the bin’s normalized Bru-seq and BruUV-seq read counts. The Viterbi algorithm was finally solved across all genome bins using a fixed transition probability of 0.005, with sequences of UVE bins taken as UVE peaks. Importantly, this process only used gene annotations to train the model, and so could detect a UVE peak at any genome location.

### Data sources (public)

All the Bru-seq and BruUV-seq data generated in this study can be found at Gene Expression Omnibus (GSE75398). Bru-seq and BruChase-seq data for HF1 was generated in our laboratory and previously published at Gene Expression Omnibus (GSE43440)^17^. The ENCODE’s long RNA-seq raw reads^52^ were downloaded from http://hgdownload.cse.ucsc.edu/goldenPath/hg19/encodeDCC/wgEncodeCshlLongRnaSeq. In order to carry out a direct comparison between Bru-seq, BruUV-seq and the ENCODE’s RNA-seq data, the ENCODE’s RNA-seq reads were trimmed to 52 base pairs and remapped as single-reads using our custom-built analysis pipeline^17^. The following K562 samples were used: whole cell poly(A)-minus replicates 1 and 2 (GEO ID: GSM758577); whole cell poly(A)-plus replicates 1 and 2 (GEO ID: GSM765405); chromatin bound replicates 3 and 4 (GEO ID: GSM765392); nucleoplasm replicates 3 and 4 (GEO ID: GSM765390); nucleus poly(A)-minus replicates 1 and 2 (GEO ID: GSM767844); nucleus poly(A)-plus replicates 1 and 2 (GEO ID: GSM765387)^53^. GRO-seq and GRO-cap data were from Gene Expression Omnibus (GEO) under accession GSE60456^18^. H3K4me3 ChIP-seq data from K562 and NHLF cells were from ENCODE (GEO: GSE29611)^54^ and visualized using the UCSC Genome Browser (http://genomes.ucsc.edu/)^55^.

## Additional Information

**How to cite this article**: Magnuson, B. *et al.* Identifying transcription start sites and active enhancer elements using BruUV-seq. *Sci. Rep.* **5**, 17978; doi: 10.1038/srep17978 (2015).

## Supplementary Material

Supplementary Information

## References

[b1] Lenhard B., Sandelin A. & Carninci P. Metazoan promoters: emerging characteristics and insights into transcriptional regulation. Nature Rev. Genetics 13, 233–245 (2012). 22392219 10.1038/nrg3163

[b2] Spitz F. & Furlong E. E. Transcription factors: from enhancer binding to developmental control. Nature Rev. Genetics 13, 613–626 (2012). 22868264 10.1038/nrg3207

[b3] Sandelin A. *et al.* Mammalian RNA polymerase II core promoters: insights from genome-wide studies. Nature Rev. Genetics 8, 424–436 (2007). 17486122 10.1038/nrg2026

[b4] Sanyal A., Lajoie B. R., Jain G. & Dekker J. The long-range interaction landscape of gene promoters. Nature 489, 109–113 (2012). 22955621 10.1038/nature11279 PMC3555147

[b5] Core L. J. *et al.* Defining the status of RNA polymerase at promoters. Cell reports 2, 1025–1035 (2012). 23062713 10.1016/j.celrep.2012.08.034 PMC3483431

[b6] Venters B. J. & Pugh B. F. Genomic organization of human transcription initiation complexes. Nature 502, 53–58 (2013). 24048476 10.1038/nature12535 PMC4018585

[b7] Shiraki T. *et al.* Cap analysis gene expression for high-throughput analysis of transcriptional starting point and identification of promoter usage. Proc. Natl. Acad. Sci. USA 100, 15776–15781 (2003). 14663149 10.1073/pnas.2136655100 PMC307644

[b8] Valen E. *et al.* Genome-wide detection and analysis of hippocampus core promoters using DeepCAGE. Genome Res. 19, 255–265 (2009). 19074369 10.1101/gr.084541.108 PMC2652207

[b9] Hashimoto S. *et al.* 5′-end SAGE for the analysis of transcriptional start sites. Nat. Biotech. 22, 1146–1149 (2004). 10.1038/nbt998 15300261

[b10] Ng P. *et al.* Gene identification signature (GIS) analysis for transcriptome characterization and genome annotation. Nature Methods 2, 105–111 (2005). 15782207 10.1038/nmeth733

[b11] Kiss D. L., Oman K., Bundschuh R. & Schoenberg D. R. Uncapped 5′ ends of mRNAs targeted by cytoplasmic capping map to the vicinity of downstream CAGE tags. FEBS Lett. 589, 279–284 (2015). 25541487 10.1016/j.febslet.2014.12.009 PMC4306605

[b12] Kim T. K. *et al.* Widespread transcription at neuronal activity-regulated enhancers. Nature 465, 182–187 (2010). 20393465 10.1038/nature09033 PMC3020079

[b13] Zentner G. E. & Scacheri P. C. The chromatin fingerprint of gene enhancer elements. J. Biol. Chem. 287, 30888–30896 (2012). 22952241 10.1074/jbc.R111.296491 PMC3438921

[b14] Hoffman M. M. *et al.* Integrative annotation of chromatin elements from ENCODE data. Nucleic Acids Res. 41, 827–841 (2013). 23221638 10.1093/nar/gks1284 PMC3553955

[b15] Andersson R. *et al.* An atlas of active enhancers across human cell types and tissues. Nature 507, 455–461 (2014). 24670763 10.1038/nature12787 PMC5215096

[b16] Core L. J., Waterfall J. J. & Lis J. T. Nascent RNA sequencing reveals widespread pausing and divergent initiation at human promoters. Science 322, 1845–1848 (2008). 19056941 10.1126/science.1162228 PMC2833333

[b17] Paulsen M. T. *et al.* Coordinated regulation of synthesis and stability of RNA during the acute TNF-induced proinflammatory response. Proc. Natl. Acad. Sci. USA 110, 2240–2245 (2013). 23345452 10.1073/pnas.1219192110 PMC3568384

[b18] Core L. J. *et al.* Analysis of nascent RNA identifies a unified architecture of initiation regions at mammalian promoters and enhancers. Nature Genetics 46, 1311–1320 (2014). 25383968 10.1038/ng.3142 PMC4254663

[b19] Kwak H., Fuda N. J., Core L. J. & Lis J. T. Precise maps of RNA polymerase reveal how promoters direct initiation and pausing. Science 339, 950–953 (2013). 23430654 10.1126/science.1229386 PMC3974810

[b20] Kruesi W. S., Core L. J., Waters C. T., Lis J. T. & Meyer B. J. Condensin controls recruitment of RNA polymerase II to achieve nematode X-chromosome dosage compensation. eLife 2, e00808 (2013). 23795297 10.7554/eLife.00808 PMC3687364

[b21] Friedberg E. *et al.* DNA Repair and Mutagenesis, Edn. Second. (ASM Press, Washington, D. C., 2006).

[b22] Tornaletti S. & Hanawalt P. Effect of DNA lesions on transcription elongation. Biochimie 81, 139–146 (1999). 10214918 10.1016/s0300-9084(99)80046-7

[b23] Donahue B. A., Yin S., Taylor J. S., Reines D. & Hanawalt P. C. Transcript cleavage by RNA polymerase II arrested by a cyclobutane pyrimidine dimer in the DNA template. Proc. Natl. Acad. Sci. USA 91, 8502–8506 (1994). 8078911 10.1073/pnas.91.18.8502 PMC44634

[b24] Sauerbier W. & Hercules K. Gene and transcription unit mapping by radiation effects. *Ann. Review* Genetics 12, 329–363 (1978). 10.1146/annurev.ge.12.120178.001553 371526

[b25] Bohr V. A., Smith C. L., Okumoto D. S. & Hanawalt P. C. DNA repair in an active gene: removal of pyrimidine dimers from the DHFR gene of CHO cells is much more efficient than in the genome overall. Cell 40, 359–369 (1985). 3838150 10.1016/0092-8674(85)90150-3

[b26] Andrade-Lima L. C., Veloso A., Paulsen M. T., Menck C. F. & Ljungman M. DNA repair and recovery of RNA synthesis following exposure to ultraviolet light are delayed in long genes. Nucleic Acids Res. 43, 2744–2756 (2015). 25722371 10.1093/nar/gkv148 PMC4357734

[b27] Paulsen M. T. *et al.* Use of Bru-Seq and BruChase-Seq for genome-wide assessment of the synthesis and stability of RNA. Methods 67, 45–54 (2014). 23973811 10.1016/j.ymeth.2013.08.015 PMC4009065

[b28] Spieth J., Brooke G., Kuersten S., Lea K. & Blumenthal T. Operons in C. elegans: polycistronic mRNA precursors are processed by trans-splicing of SL2 to downstream coding regions. Cell 73, 521–532 (1993). 8098272 10.1016/0092-8674(93)90139-h

[b29] Blumenthal T. Operons in eukaryotes. Brief. Funct. Genomics & Proteomics 3, 199–211 (2004). 10.1093/bfgp/3.3.199 15642184

[b30] Dieci G., Preti M. & Montanini B. Eukaryotic snoRNAs: a paradigm for gene expression flexibility. Genomics 94, 83–88 (2009). 19446021 10.1016/j.ygeno.2009.05.002

[b31] Ren S. *et al.* RNA-seq analysis of prostate cancer in the Chinese population identifies recurrent gene fusions, cancer-associated long noncoding RNAs and aberrant alternative splicings. Cell Res. 22, 806–821 (2012). 22349460 10.1038/cr.2012.30 PMC3343650

[b32] Tomlins S. A. *et al.* Recurrent fusion of TMPRSS2 and ETS transcription factor genes in prostate cancer. Science 310, 644–648 (2005). 16254181 10.1126/science.1117679

[b33] Kumar-Sinha C., Tomlins S. A. & Chinnaiyan A. M. Evidence of recurrent gene fusions in common epithelial tumors. Trends Mol. Med. 12, 529–536 (2006). 17011825 10.1016/j.molmed.2006.09.005

[b34] Daley G. Q., Van Etten R. A. & Baltimore D. Induction of chronic myelogenous leukemia in mice by the P210bcr/abl gene of the Philadelphia chromosome. Science 247, 824–830 (1990). 2406902 10.1126/science.2406902

[b35] Whyte W. A. *et al.* Master transcription factors and mediator establish super-enhancers at key cell identity genes. Cell 153, 307–319 (2013). 23582322 10.1016/j.cell.2013.03.035 PMC3653129

[b36] Parker S. C. *et al.* Chromatin stretch enhancer states drive cell-specific gene regulation and harbor human disease risk variants. Proc. Natl. Acad. Sci. USA 110, 17921–17926 (2013). 24127591 10.1073/pnas.1317023110 PMC3816444

[b37] Lubas M. *et al.* The human nuclear exosome targeting complex is loaded onto newly synthesized RNA to direct early ribonucleolysis. Cell Reports 10, 178–192 (2015). 25578728 10.1016/j.celrep.2014.12.026

[b38] Blasius M., Wagner S.A., Choudhary C., Bartek J. & Jackson S.P. A quantitative 14-3-3 interaction screen connects the nuclear exosome targeting complex to the DNA damage response. Genes & Dev. 28, 1977–1982 (2014). 25189701 10.1101/gad.246272.114 PMC4173157

[b39] Tiedje C. *et al.* p38MAPK/MK2-mediated phosphorylation of RBM7 regulates the human nuclear exosome targeting complex. RNA 21, 262–278 (2015). 25525152 10.1261/rna.048090.114 PMC4338353

[b40] Veloso A. *et al.* Rate of elongation by RNA polymerase II is associated with specific gene features and epigenetic modifications. Genome Res. 24, 896–905 (2014). 24714810 10.1101/gr.171405.113 PMC4032854

[b41] Rockx D. A. *et al.* UV-induced inhibition of transcription involves repression of transcription initiation and phosphorylation of RNA polymerase II. Proc. Natl. Acad. Sci. USA 10, 10503–10508 (2000). 10973477 10.1073/pnas.180169797 PMC27054

[b42] Thurman R. E. *et al.* The accessible chromatin landscape of the human genome. Nature 489, 75–82 (2012). 22955617 10.1038/nature11232 PMC3721348

[b43] Li G. *et al.* Extensive promoter-centered chromatin interactions provide a topological basis for transcription regulation. Cell 148, 84–98 (2012). 22265404 10.1016/j.cell.2011.12.014 PMC3339270

[b44] Ljungman M. & Zhang F. Blockage of RNA polymerase as a possible trigger for u.v. light-induced apoptosis. Oncogene 13, 823–831 (1996). 8761304

[b45] Veloso A. *et al.* Genome-wide transcriptional effects of the anti-cancer agent camptothecin. PloS One 8, e78190 (2013). 24194914 10.1371/journal.pone.0078190 PMC3806802

[b46] Langmead B., Trapnell C., Pop M. & Salzberg S. L. Ultrafast and memory-efficient alignment of short DNA sequences to the human genome. Genome Biol. 10, R25 (2009). 19261174 10.1186/gb-2009-10-3-r25 PMC2690996

[b47] Trapnell C., Pachter L. & Salzberg S. L. TopHat: discovering splice junctions with RNA-Seq. Bioinformatics 25, 1105–1111 (2009). 19289445 10.1093/bioinformatics/btp120 PMC2672628

[b48] Pruitt K. D. *et al.* RefSeq: an update on mammalian reference sequences. Nucleic Acids Res. 42, D756–763 (2014). 24259432 10.1093/nar/gkt1114 PMC3965018

[b49] Flicek P. *et al.* Ensembl 2013. Nucleic Acids Res. 41, D48–55 (2013). 23203987 10.1093/nar/gks1236 PMC3531136

[b50] Jackson C. Multis-state models for panel data: the msm package for R. J. Statistical Software 38, 1–29 (2011).

[b51] Maechler M., Rousseeuw P., Struyf A., Hubert M. & Hornik K. cluster: Cluster Analysis Basics and Extensions. R package version 1.14.4. (2013).

[b52] Bernstein B. E. *et al.* An integrated encyclopedia of DNA elements in the human genome. Nature 489, 57–74 (2012). 22955616 10.1038/nature11247 PMC3439153

[b53] Tilgner H. *et al.* Deep sequencing of subcellular RNA fractions shows splicing to be predominantly co-transcriptional in the human genome but inefficient for lncRNAs. Genome Res. 22, 1616–1625 (2012). 22955974 10.1101/gr.134445.111 PMC3431479

[b54] Consortium E. P. An integrated encyclopedia of DNA elements in the human genome. Nature 489, 57–74 (2012). 22955616 10.1038/nature11247 PMC3439153

[b55] Kent W. J. *et al.* The human genome browser at UCSC. Genome Res. 12, 996–1006 (2002). 12045153 10.1101/gr.229102 PMC186604

