# Supplementary material for: Identifying transcription start sites and active enhancer elements using BruUV-seq
Source: Sci Rep. 2015 Dec 11;5:17978. doi: 10.1038/srep17978 (PMC4675984; doi:10.1038/srep17978)
Supplement: Supplementary Information [file srep17978-s1.pdf]

Supplementary Material

Identifying transcription start sites and active enhancer elements using BruUV-seq

Brian Magnuson, Artur Veloso, Killeen S. Kirkconnell, Leonardo Carmo de Andrade Lima, Michelle T. Paulsen, Emily A. Ljungman, Karan Bedi, Jayendra Prasad, Thomas E. Wilson and Mats Ljungman

Table of contents:

Supplementary Figures 1-10

Supplementary Table 1

Supplementary Material

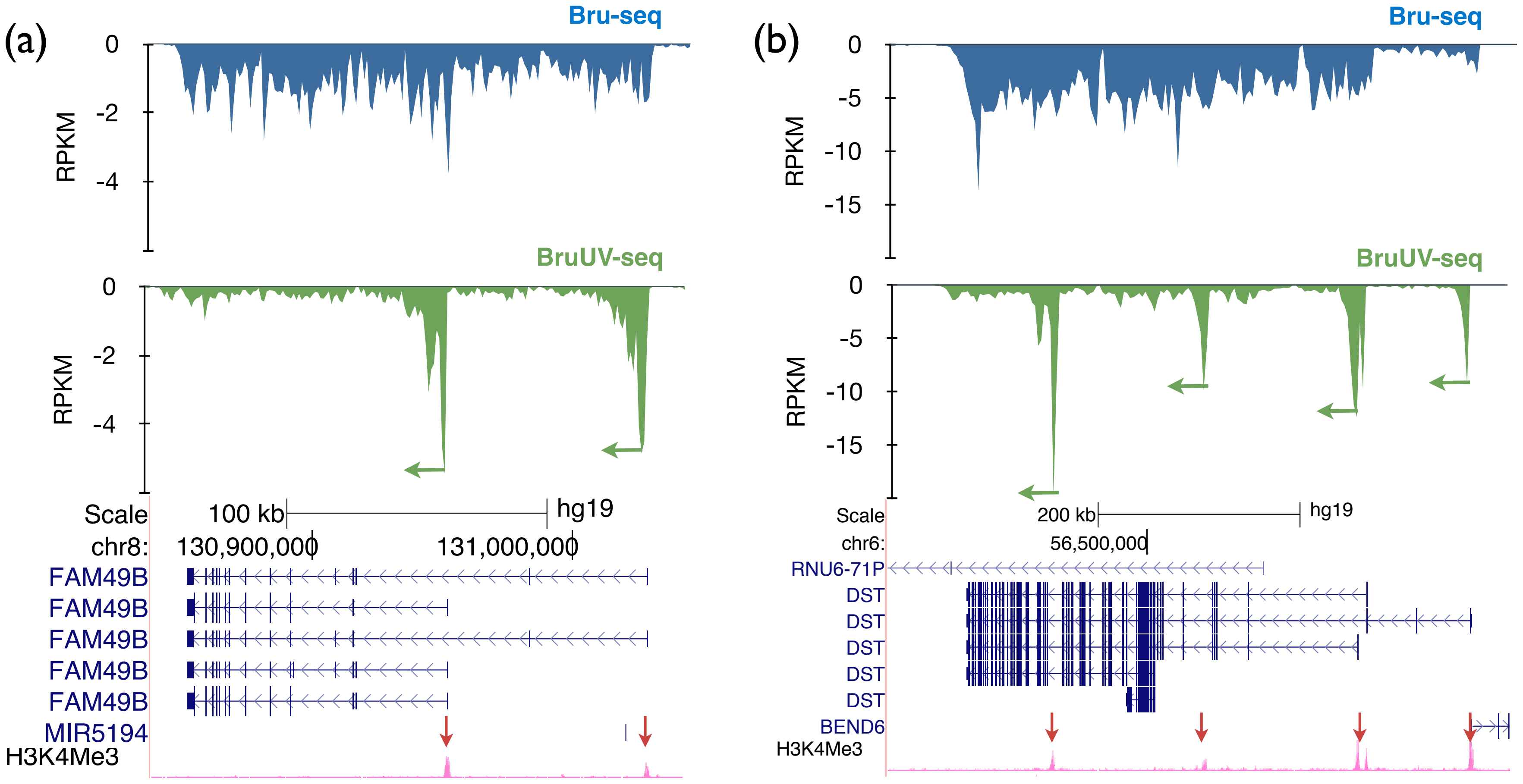

Supplementary Figure 1. BruUV-seq identifies multiple TSSs. Human fibroblasts HF1 were subjected to Bru-seq (top) or BruUV-seq (bottom). (*a*) The *FAM49B* gene is transcribed from two major TSSs which are lining up with the two major H3K4me3 peaks (red arrows). (*b*) The *DST* gene initiates transcription from 4 different TSSs that align with H3K4me3 peaks. The gene maps are from RfSeq and the H3K4me3 data from ENCODE (UCSC web browser).

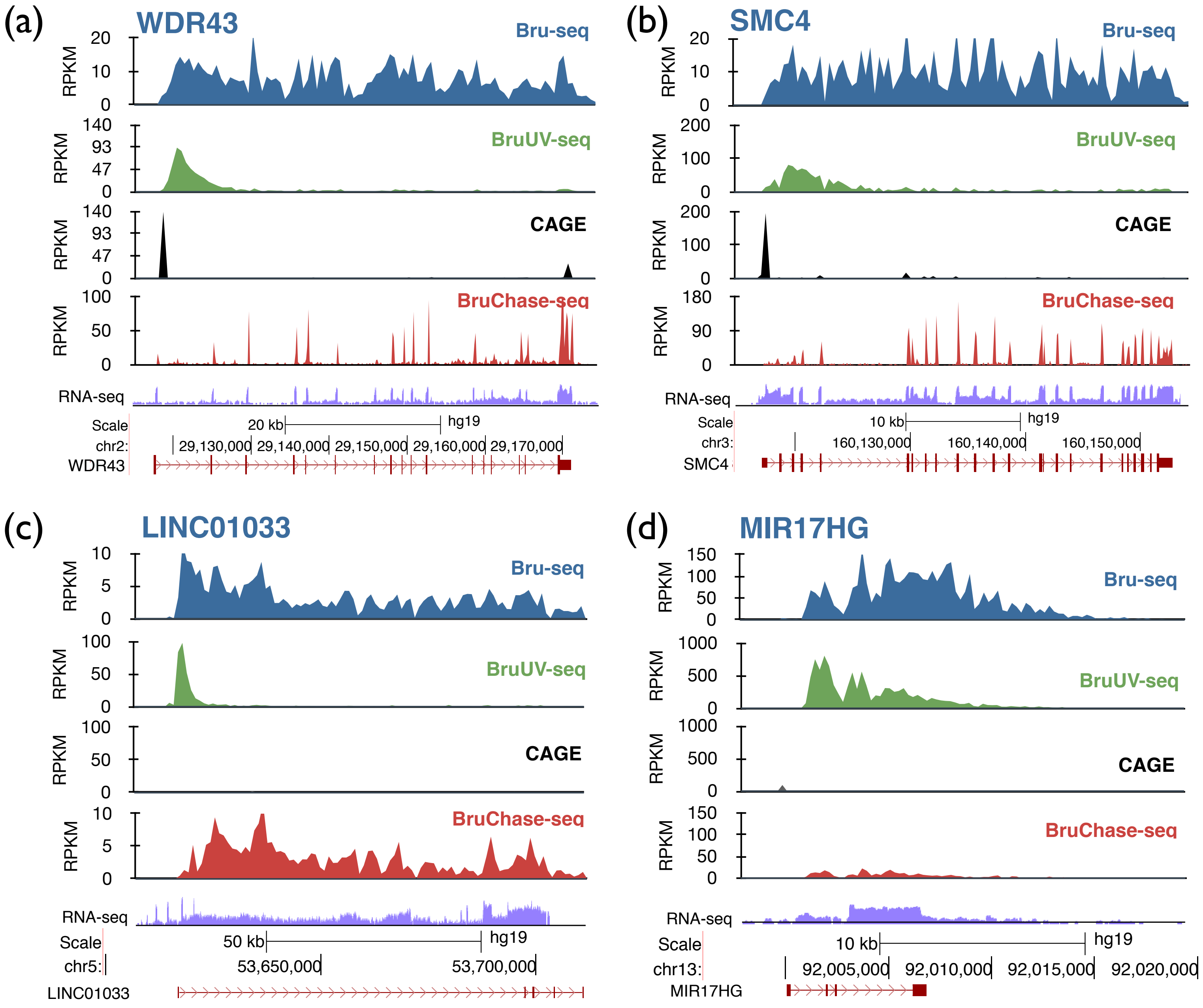

Supplementary Figure 2. Comparison of BruUV-seq and CAGE in K562 cells. (*a*) WDR43, (*b*) SMC4, (*c*) LINC01033 and (*d*) MIR17HG genes were analyzed and their signatures of Bru-seq (blue), BruUV-seq (green), CAGE (black) and BruChase-seq (red) are shown. The RNA-seq data is from ENCODE and the gene maps are from ENSAMBLE obtained from the UCSC web browser.

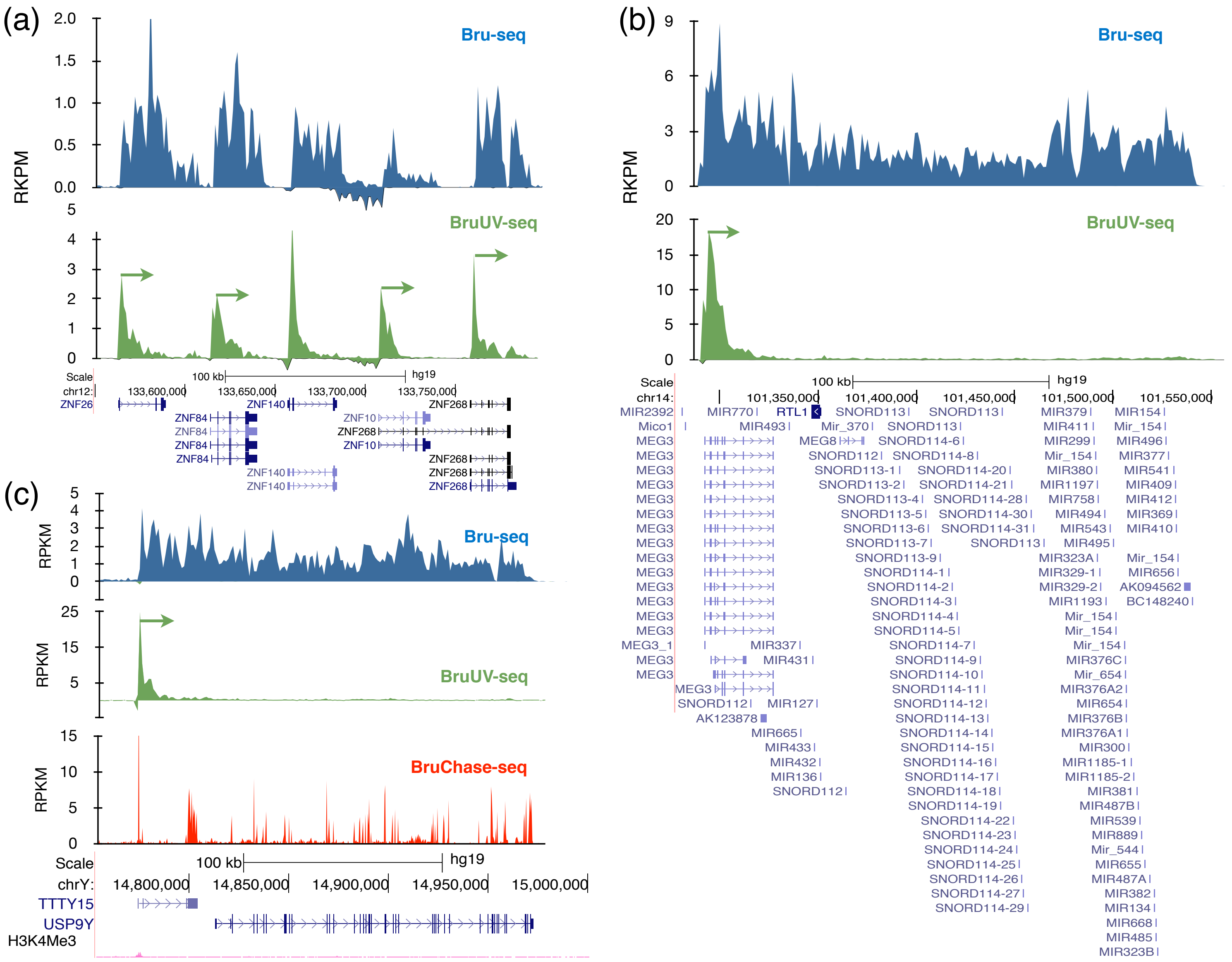

Supplementary Figure 3. BruUV-seq distinguishes between gene clusters initiating from individual or common promoters in HF1 cells. (**a**) Bru-seq (blue) and BruUV-seq (green) data of genes encoding the zinc-finger proteins *ZNF26, ZNF84, ZNF140, ZNF10* and *ZNF268* on chromosome 12 are shown. Each gene uses their individual TSS to direct their transcription. (**b**) Bru-seq and BruUV-seq data of a gene cluster on chromosome 14 transcribing as an operon from a common TSS. (**c**) Bru-seq, BruUV-seq and BruChase (6 h chase) data for the *TTTY15* and *USP9Y* genes transcribing as an operon from a common TSS. Note that the primary transcript appears to be spliced. The gene maps are from RefSeq taken from the UCSC web browser.

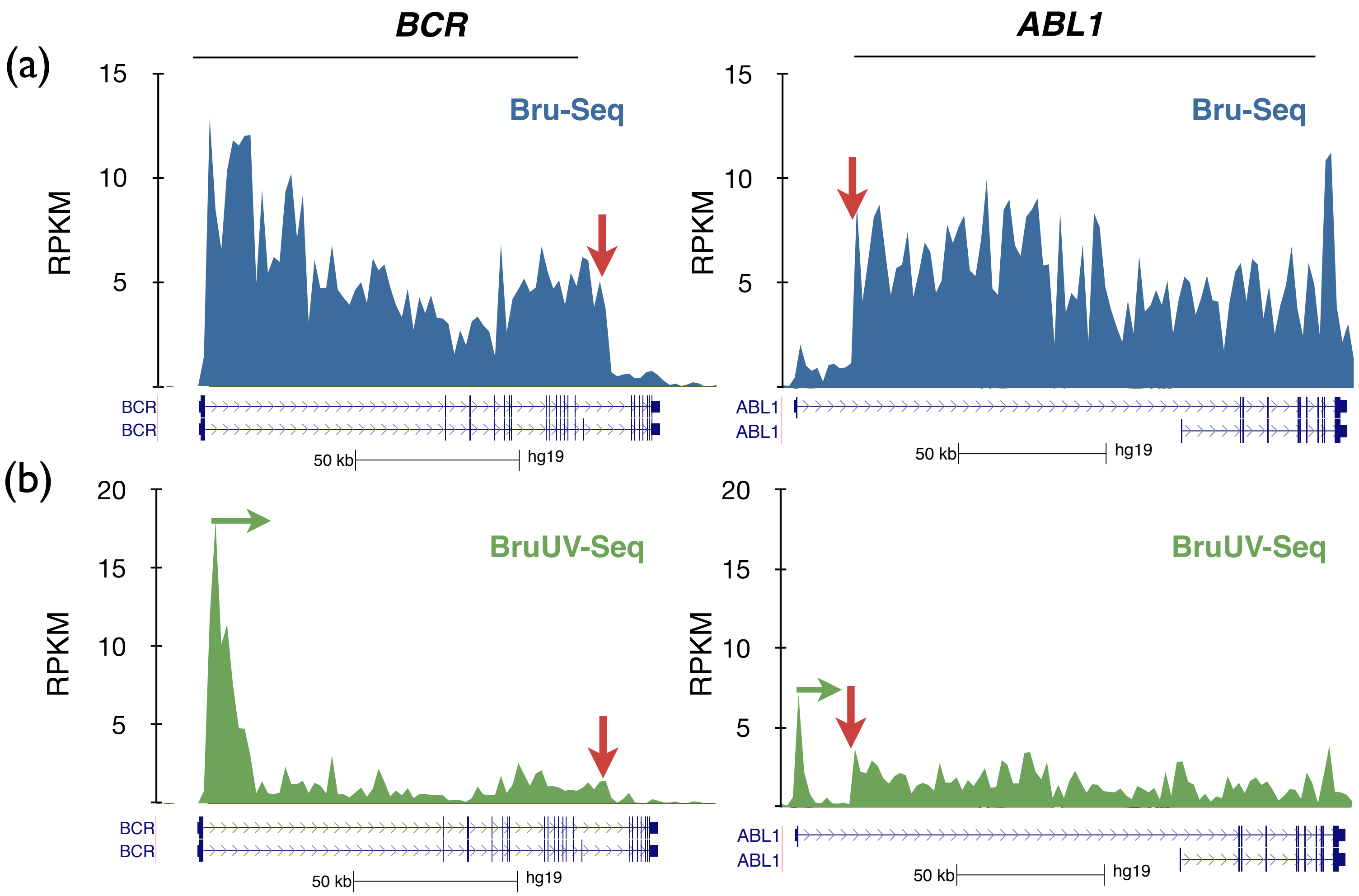

Supplementary Figure 4. BruUV-seq verifies the presence of the *BCR-ABL* fusion gene in K562 leukemia cells. (*a*) Nascent transcription (Bru-seq) is high in the *BCR* gene up to the breakpoint (arrow) where the transcription reads drop off. In contrast to the *BCR* gene, transcription from the *ABL1* gene (right) starts off with low counts until the breakpoint where the reads jump up. (*b*) Using BruUV-seq we observe the strong TSS in the *BCR* gene (left) and a small in the *ABL1* gene from the unaffected allele while no UV peak is observed at the breakpoint verifying that the increased downstream transcription must initiate elsewhere (at the TSS of the *BCR* gene).

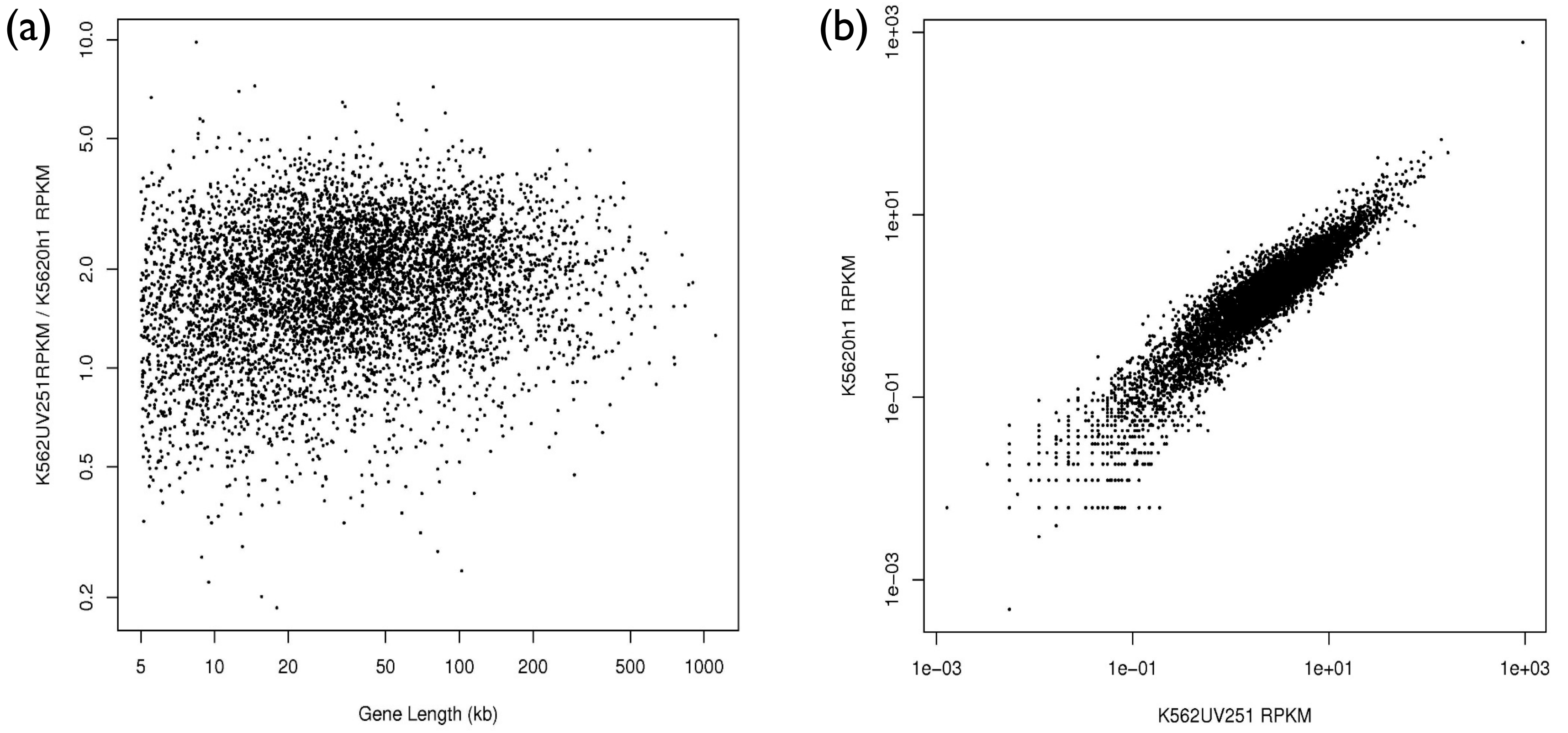

Supplementary Figure 5. UV-mediated inhibition does not affect the ratio between BruUV-seq and Bru-seq in the gene's first 5kb. (*a*) Absence of correlation between UV-mediated inhibition of transcription and genesize when measuring signal in the first 5kb of genes. The ratio of integrated transcription reads following UV-irradiation in HF1 human fibroblasts compared to mock-irradiated K562 cells are shown on the Y-axis while gene size is shown on the X-axis. (*b*) Positive correlation between gene expression (measured in the first 5kb of the gene) between UV-irradiated (X-axis) and mock-irradiated (Y-axis) K562 cells.

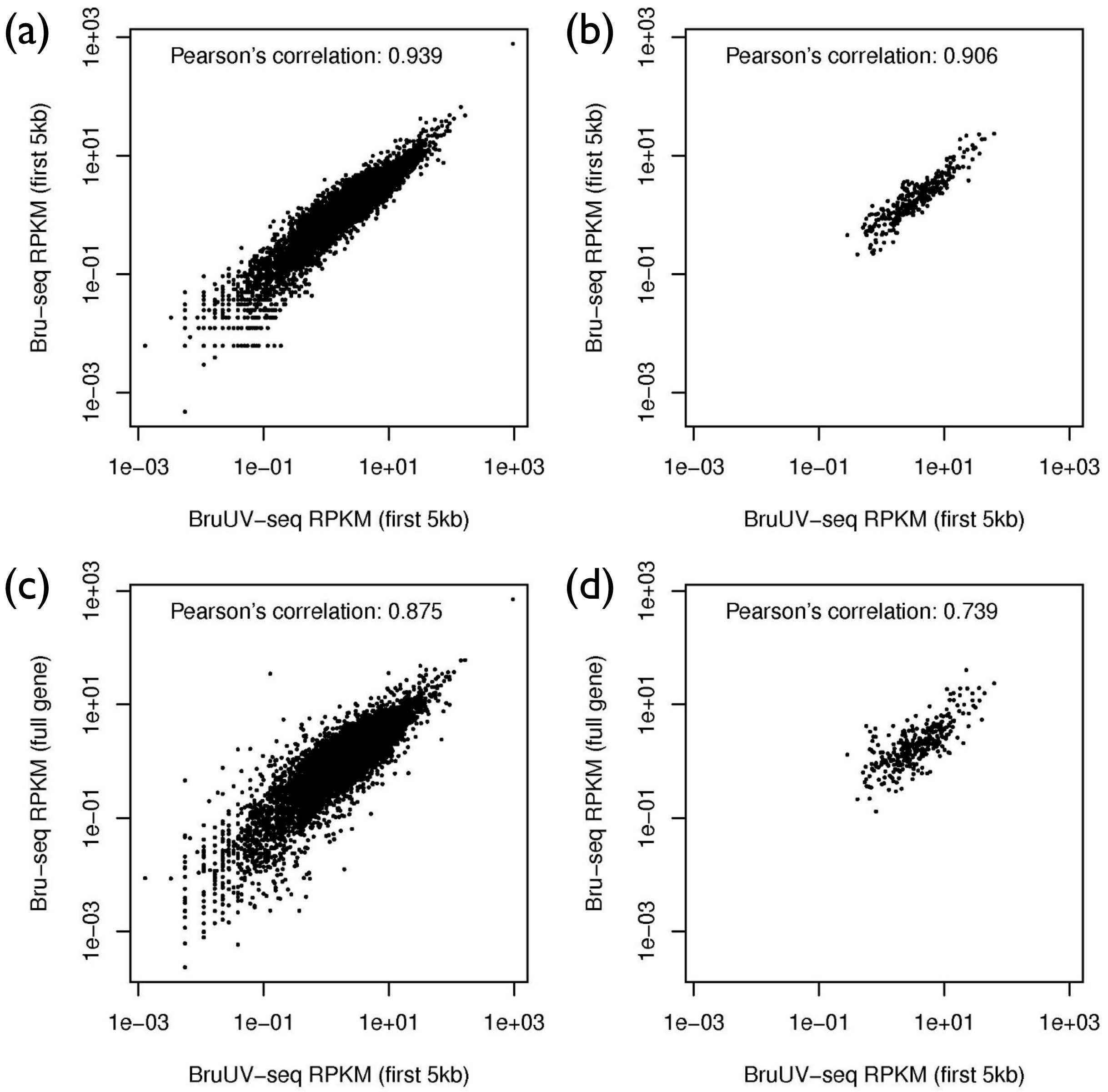

Supplementary Figure 6. Supp Fig 5. BruUV-seq expression signal in the first 5kb of genes is correlated to gene expression in mock-irradiated cells. Geneexpression levels following UV-irradiation were measured in the first 5kb of genes and are displayed in the X-axis (*a-d*). Bru-seq calculated expression values measured based on the first 5kb (*a,b*) or whole gene (*c,d*) are displayed in the Y-axis. The panels have been split in order to differentiate between genes with one active TSS (*a,c*) and two or more active TSS (*b,d*).

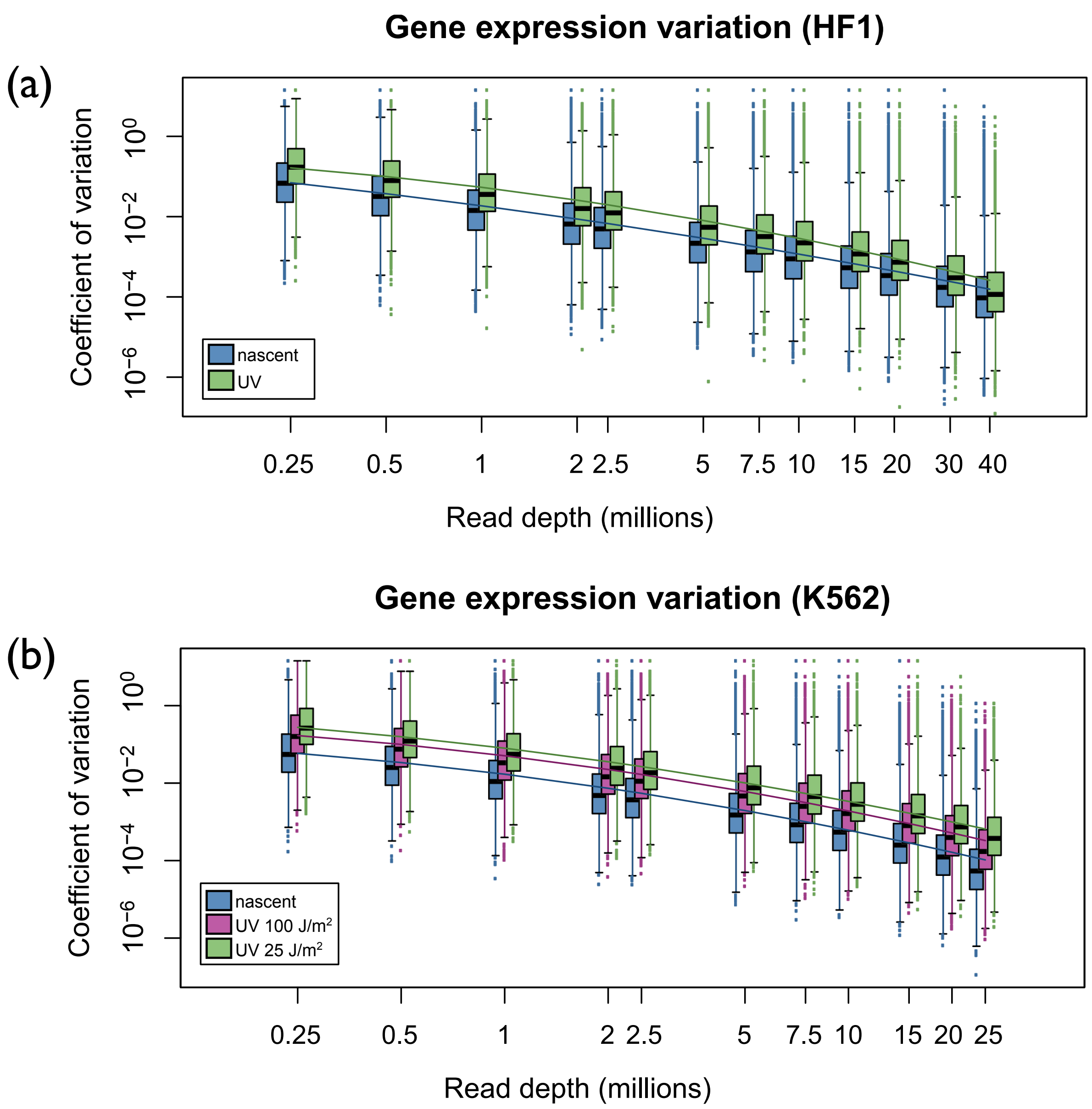

Supplementary Figure 7. Performance of BruUV-seq vs. Bru-seq in determining gene expression values at various read depths. (*a*) Variation of gene expression calculated from a BruUV-seq sample is higher, but similar to, values calculated from Bru-seq samples at various simulated read depths in HF1 cells. (*b*) In K562 cells, the similarity in coefficients of variation between Bru-seq- and BruUV-seq-derived data is dependent on UV dose. In both (*a*) and (*b*), gene expression (RPKM) was calculated at indicated read depths by randomly sampling experimental data 10 times each. Coefficients of variation (standard deviation divided by mean) were determined per gene from these 10 RPKM values. For BruUV-seq samples, only the first 5kb of genes was used to calculate RPKM.

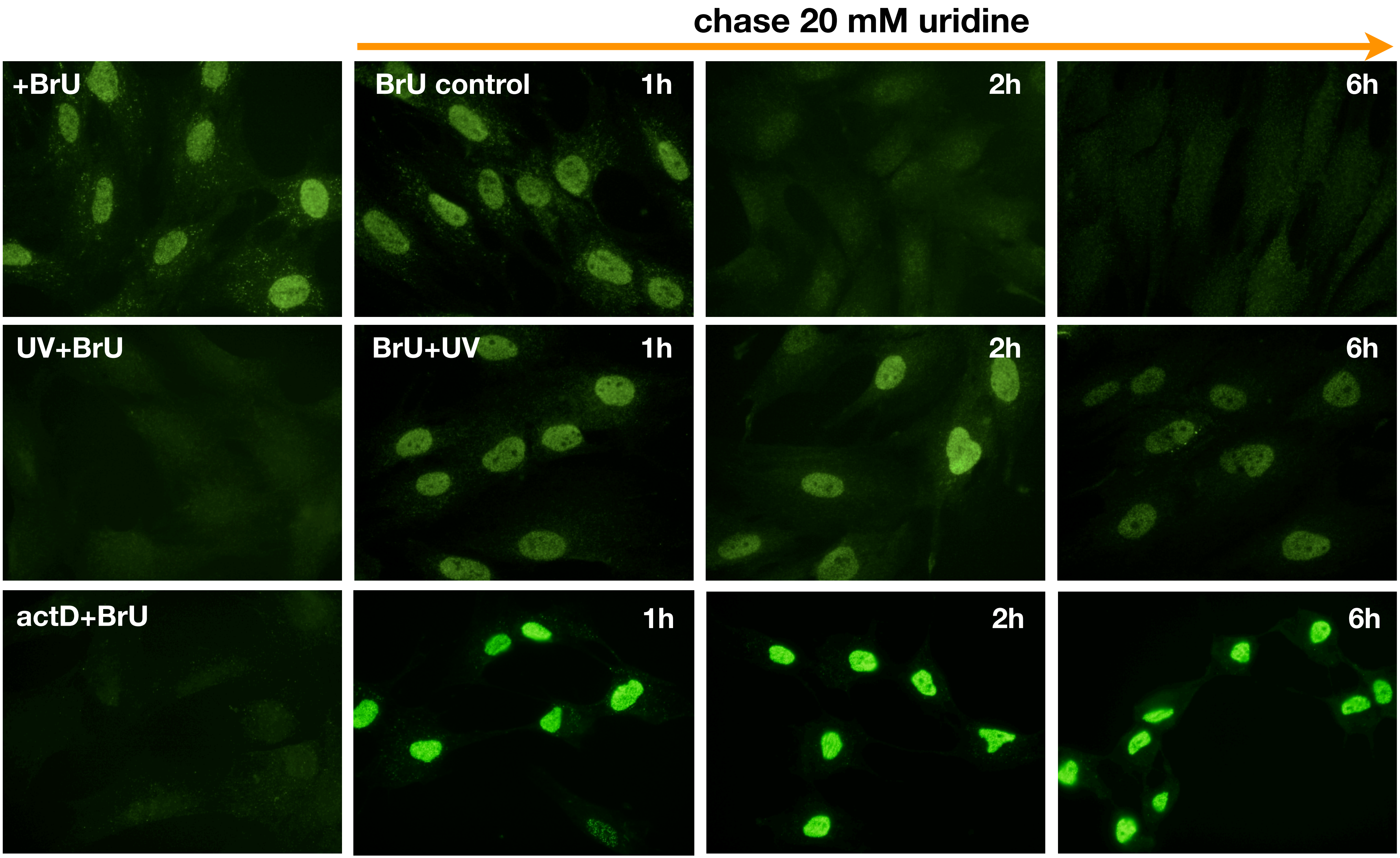

Supplementary Figure 8. Bromouridine staining in human fibroblasts HF1. Top row: Cells were labeled for 30 min with 2 mM Bru followed by chase in 20 mM uridine for different times. Middle row: Cells were irradiated with 50 J/m2 directly prior (left) or following Bru-labeling, followed by uridine chase. Bottom row: Cells were treated with 10 µg/ml actinomycin D during Bru-labeling (left) or following labeling, during the chase in uridine.

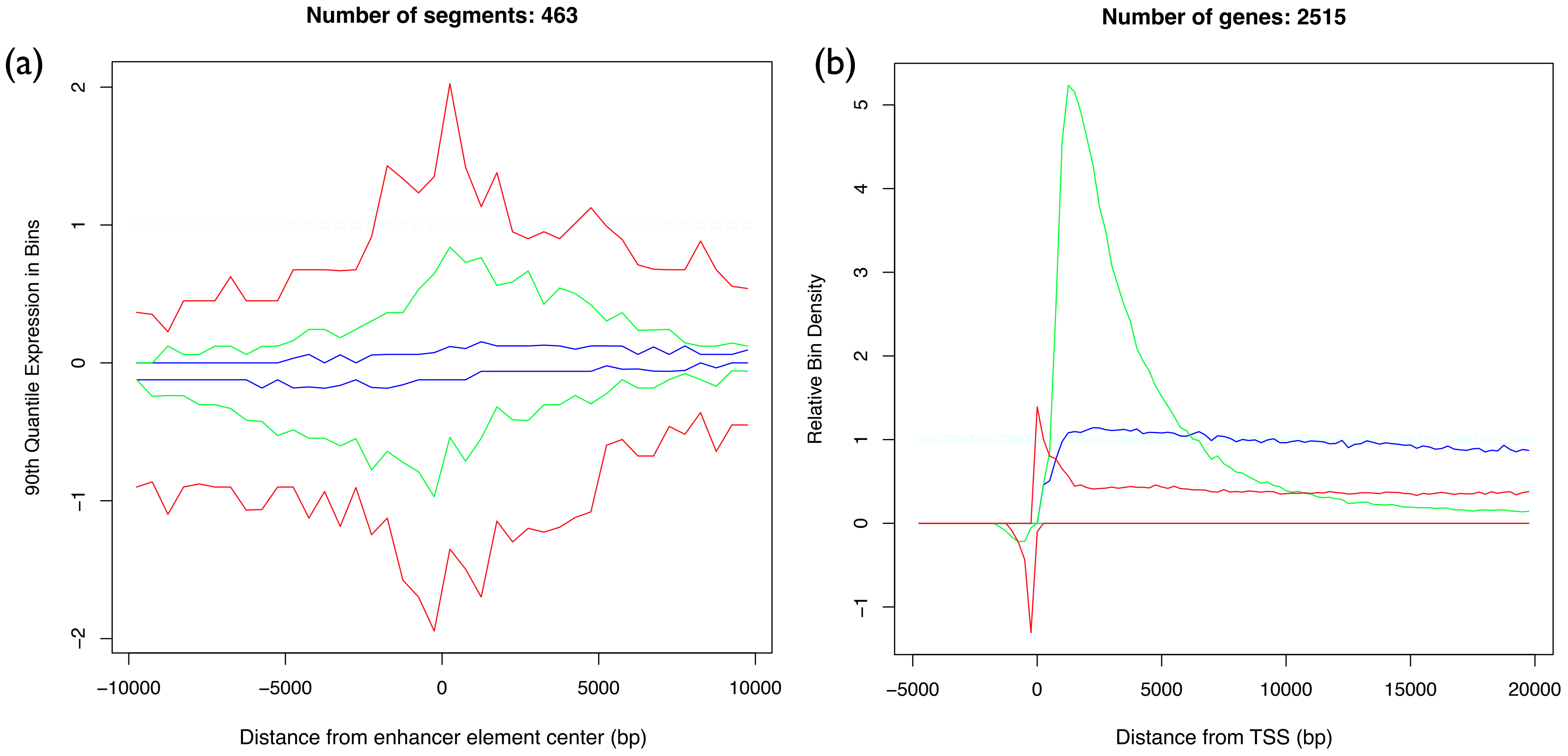

Supplementary Figure 9. Comparison of nascent RNA signal from K562 cells between Bru-seq (blue), BruUV-seq (100 J/m^2; green), and GRO-seq (red) in (a) ENCODE-classified intergenic enhancers and (b) TSS of expressed RefSeq genes. In (a), enhancers were selected if nascent RNA segments were identified in both BruUV-seq and GRO-seq (463 segments). Genes represented in (b) were at least 20 kb long and expressed in the Bru-seq sample (2515 genes).

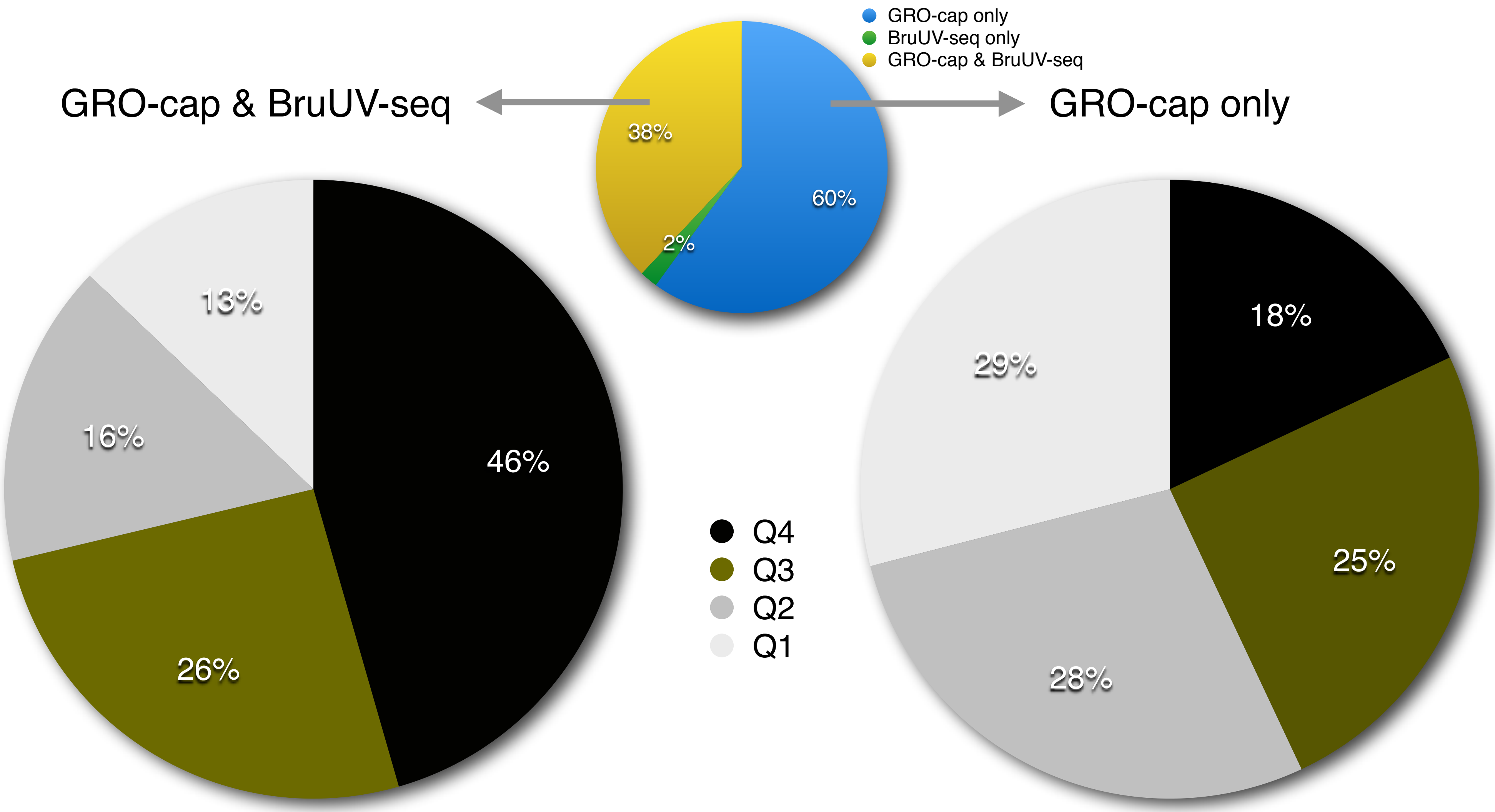

Supplementary Figure 10. Comparison of GRO-cap signal in ENCODE enhancer elements from Fig 4f (small pie chart, top) where signal was found for both GRO-cap and BruUV-seq (left) and for signal detected only by GRO-cap (right). The GRO-cap signal is divided into four intensity quartiles where Q1 is the lowest (light shade) and Q4 is the highest intensity (dark shade). It can be seen that enhancer identified by BruUV-seq correspond to more GRO-cap signals with high intensity (dark shade) compared with enhancer elements identified by GRO-cap alone.

chrom	start	end	name	strand	bp	meanRPKM	meanCount	TNF	control	foldChange	BruUV peaks
chr4	74606222	74609433	IL8	+	3211	81.857	12111	24147	75	320.439	**>10**
chr4	74735108	74736955	CXCL1	+	1847	37.148	3161	6302	20	306.648	**1**
chr4	74902311	74904490	CXCL3	-	2179	9.596	963	1918	7	248.939	**1**
chr6	31543349	31546112	TNF	+	2763	3.138	399	795	3	232.159	**>10**
chr4	74962753	74964997	CXCL2	-	2244	21.388	2209	4394	24	176.944	**>5**
chr19	10381516	10397291	ICAM1	+	15775	4.284	3110	6182	38	160.424	**0**
chr2	113587336	113594356	IL1B	-	7020	16.369	5285	10486	84	123.691	**>5**
chr6	160100148	160114353	SOD2	-	14205	35.869	23427	46428	426	108.869	**2**
chr17	77020250	77045870	C1QTNF1	+	25620	2.163	2546	5040	53	94.927	**4**
chr2	113531491	113542971	IL1A	-	11480	7.951	4193	8288	97	84.904	**>5**
chr17	32582295	32584220	CCL2	+	1925	13.216	1168	2306	29	76.948	**>10**
chr9	123664670	123691451	TRAF1	-	26781	2.122	2606	5125	87	58.676	**5**
chr3	157154579	157161417	PTX3	+	6838	58.669	18394	36166	621	58.172	**0**
chr8	125563027	125740730	MTSS1	-	177703	2.487	20230	39583	876	45.14	**>5**
chr8	72753776	72756731	MSC	-	2955	0.861	116	226	5	37.792	**2**
chr7	22766765	22771621	IL6	+	4856	6.219	1380	2688	71	37.37	**5**
chr15	45722762	45725647	C15orf48	+	2885	0.741	97	190	5	37.045	**2**
chr17	9548949	9633003	USP43	+	84054	0.744	2857	5554	160	34.689	**0**
chr6	14117486	14137148	CD83	+	19662	0.776	696	1353	40	33.627	**0**
chr19	45504706	45541456	RELB	+	36750	1.462	2452	4758	146	32.496	**0**
chr6	138188580	138204449	TNFAIP3	+	15869	8.871	6418	12420	416	29.843	**>5**
chr21	34602230	34636820	IFNAR2	+	34590	6.351	10009	19326	691	27.931	**2**
chr5	131817300	131826465	IRF1	-	9165	6.024	2513	4844	182	26.562	**2**
chr14	103592663	103603776	TNFAIP2	+	11113	3.787	1915	3691	140	26.283	**0**
chr3	189674516	189840226	LEPREL1	-	165710	10.692	80586	154837	6335	24.441	**>5**
chr3	10206562	10285427	IRAK2	+	78865	2.81	10076	19346	807	23.957	**5**
chr5	118604417	118730294	TNFAIP8	+	125877	2.708	15478	29604	1353	21.88	**4**
chr21	26934456	26947480	MIR155HG	+	13024	33.869	20018	38220	1817	21.033	**1**
chr15	71184927	71342436	LRRC49	+	157509	2.14	15272	29017	1526	19.004	**0**
chr7	134127106	134143888	AKR1B1	-	16782	3.72	2825	5352	298	17.962	**0**
chr10	75670861	75677258	PLAU	+	6397	1.3	376	711	41	17.301	**>5**
chr4	113196781	113207059	TIFA	-	10278	2.932	1362	2574	150	17.084	**0**
chr11	10471867	10529126	AMPD3	+	57259	2.504	6477	12206	749	16.29	**5**
chr1	205271190	205290883	NUAK2	-	19693	7.306	6500	12246	754	16.232	**5**
chr1	89573309	89591799	GBP2	-	18490	1.494	1247	2343	150	15.55	**0**
chr8	22224761	22291640	SLC39A14	+	66879	4.081	12310	23084	1536	15.026	**2**
chr1	110453232	110473616	CSF1	+	20384	5.656	5200	9749	650	14.98	**1**
chr2	111878490	111926022	BCL2L11	+	47532	0.821	1757	3280	234	13.98	**0**
chr12	10310898	10324790	OLR1	-	13892	1.904	1191	2222	160	13.877	**>10**
chr14	96722546	96731100	BDKRB1	+	8554	16.129	6210	11571	849	13.621	**3**
chr7	41728600	41742706	INHBA	-	14106	18.433	11694	21738	1651	13.167	**2**
chr6	143072603	143266338	HIVEP2	-	193735	8.578	74672	138405	10939	12.652	**>5**
chr22	18216905	18257431	BID	-	40526	0.75	1366	2531	201	12.58	**0**
chr14	35870715	35873960	NFKBIA	-	3245	25.251	3677	6793	561	12.094	**0**
chr12	50031723	50101197	FMNL3	-	69474	2.701	8421	15545	1297	11.982	**1**
chr6	126102306	126253176	NCOA7	+	150870	2.918	19748	36424	3072	11.855	**5**
chr8	23536205	23540450	NKX3-1	-	4245	1.333	253	464	41	11.076	**0**
chr12	105197274	105322472	SLC41A2	-	125198	0.94	5270	9658	882	10.95	**1**
chr21	34775201	34809828	IFNGR2	+	34627	4.449	6894	12623	1165	10.831	**3**
chr10	104154228	104162281	NFKB2	+	8053	0.616	222	406	37	10.785	**0**
chr8	124510126	124553493	FBXO32	-	43367	3.094	6005	10988	1021	10.756	**1**
chr5	150409503	150467221	TNIP1	-	57718	4.624	11926	21732	2119	10.254	**1**
chr6	12290528	12297427	EDN1	+	6899	3.858	1189	2165	213	10.153	**4**
chr4	103422485	103538459	NFKB1	+	115974	9.379	48565	88266	8864	9.957	**2**
chr8	23386362	23430063	SLC25A37	+	43701	3.248	6336	11513	1159	9.93	**1**
chr8	72755357	72968547	LOC100132891	+	213190	0.747	7108	12896	1321	9.76	**1**
chr11	102706527	102714342	MMP3	-	7815	3.007	1048	1902	195	9.743	**5**

chr2	224839764	224904036	SERPINE2	-	64272	2.192	6269	11266	1271	8.86	**0**
chr2	68694690	68807294	APLF	+	112604	0.587	2939	5281	597	8.836	**0**
chr6	44225902	44233525	NFKBIE	-	7623	0.855	289	519	59	8.669	**0**
chr1	218518675	218617961	TGFB2	+	99286	2.086	9206	16508	1905	8.664	**4**
chr11	34172533	34379555	ABTB2	-	207022	0.722	6639	11892	1386	8.578	**0**
chr12	91497231	91505542	LUM	-	8311	9.727	3590	6419	762	8.422	**>10**
chr12	102271104	102317401	DRAM1	+	46297	14.08	28940	51655	6225	8.297	**3**
chr4	38665789	38703129	KLF3	+	37340	13.769	22814	40659	4969	8.182	**1**
chr2	206547223	206662857	NRP2	+	115634	4.397	22556	40169	4942	8.127	**3**
chr6	32812985	32821748	TAP1	-	8763	0.923	358	638	78	8.108	**0**
chr14	71374121	71582099	PCNX	+	207978	7.527	69435	123589	15281	8.088	**3**
chr9	71736223	71870124	TJP2	+	133901	1.331	7903	14066	1740	8.08	**0**
chr17	40439564	40463960	STAT5A	+	24396	0.524	566	1007	125	8.006	**0**
chr10	47133294	47133836	LOC728643	-	542	1.891	45	80	10	7.841	**0**
chr10	70847827	70864567	SRGN	+	16740	1.765	1309	2321	297	7.812	**0**
chr3	194406621	194409766	FAM43A	+	3145	3.143	437	774	101	7.662	**0**
chr12	26272958	26278003	BHLHE41	-	5045	1.257	280	496	65	7.626	**3**
chr2	61108751	61150178	REL	+	41427	1.986	3639	6419	860	7.459	**0**
chr17	67240575	67323323	ABCA5	-	82748	2.455	8976	15784	2168	7.28	**0**
chr3	11178778	11304939	HRH1	+	126161	3.074	17118	29980	4256	7.043	**0**
chr3	100468178	100712334	ABI3BP	-	244156	0.6	6456	11281	1630	6.919	**>5**
chr10	30722949	30750762	MAP3K8	+	27813	1.359	1666	2906	426	6.815	**0**
chr14	77253585	77279283	ANGEL1	-	25698	0.842	953	1660	245	6.756	**0**
chr2	159825145	160089170	TANC1	+	264025	3.675	42753	74463	11043	6.743	**1**
chr6	37137921	37143204	PIM1	+	5283	2.487	578	1007	149	6.725	**3**
chr1	209848669	209849735	G0S2	+	1066	3.313	155	269	41	6.562	**2**
chr19	45251977	45263301	BCL3	+	11324	1.079	537	931	143	6.516	**4**
chr11	102217965	102249401	BIRC2	+	31436	10.255	14175	24521	3829	6.403	**0**
chr15	45927255	45983479	SQRDL	+	56224	1.43	3534	6104	963	6.337	**1**
chr8	22298482	22398657	PPP3CC	+	100175	4.256	18735	32344	5126	6.309	**1**
chr10	95653729	95662491	SLC35G1	+	8762	1.758	676	1165	187	6.214	**1**
chr12	91539034	91576806	DCN	-	37772	0.699	1159	1996	322	6.185	**5**
chr3	5021096	5026865	BHLHE40	+	5769	2.519	637	1097	178	6.163	**3**
chr4	75230859	75254477	EREG	+	23618	7.267	7530	12926	2133	6.06	**0**
chr3	106959538	107045811	LOC344595	+	86273	0.778	2942	5048	836	6.034	**4**
chr18	55711609	56068772	NEDD4L	+	357163	2.641	41364	70912	11816	6.001	**3**
chr1	117057155	117113715	CD58	-	56560	1.743	4321	7394	1249	5.918	**0**
chr21	33784751	33887697	C21orf63	+	102946	0.999	4508	7712	1303	5.918	**0**
chr1	120161999	120190390	ZNF697	-	28391	2.021	2514	4299	728	5.9	**1**
chr9	117781853	117880536	TNC	-	98683	2.239	9679	16544	2814	5.878	**2**
chr14	55034329	55260033	SAMD4A	+	225704	13.161	130113	222180	38046	5.84	**4**
chr8	67474409	67525480	MYBL1	-	51071	6.013	13451	22968	3934	5.837	**>5**

Supplementary Table I.

Number of TNF-induced BruUV-seq peaks associated with the the top 100 genes induced in HF1 cells following a 1 hr treatment with TNF.
Orange fields: genes associated with >5 TNF-induced BruUV-seq peaks;
Green fields: TNF-induced genes with no TNF-induced BruUV-seq peaks.

SUPPLEMENTARY MATERIAL

BruUV-seq and Bru-seq signals are positively correlated

Gene length influences the difference between gene expression values measured by Bru-seq and BruUV-seq (Fig. 1b). We reasoned that this bias would be eliminated by using only a short region downstream of the TSS for measuring expression. Indeed, the ratio between Bru-seq and BruUV-seq signal in the first 5 kb of genes was not correlated to the sizes of the genes (Supplementary Fig. 4a). Furthermore, the Bru-seq and BruUV-seq expression measurements within the first 5 kb of the genes were strongly correlated (Supplementary Fig. 4b, r=0.939). The BruUV-seq signal in the first 5 kb after the TSS was also correlated with Bru-seq expression along the whole length of the genes, albeit more weakly (Supplementary Fig. 5c, r=0.875). This difference was more pronounced for genes expressing two or more TSS (Supplementary Fig. 5d, r=0.739). The positive correlation between the two techniques suggests that BruUV-seq data are predictive of Bru-seq data and therefore BruUV-seq could be used as a surrogate for nascent RNA transcription measurements with Bru-seq.

Using a restricted portion of a gene to calculate its expression value, however, is counterintuitive since one generally wants to use all available data. To determine if restricting the expression measurement was reasonable, we compared the amount of variation observed in both approaches. We randomly selected a given number of reads 10 times from both Bru-seq and BruUV-seq samples to simulate a given read depth. The median coefficient of variation in gene expression from Bru-seq and BruUV-seq were very similar, but consistently lower in Bru-seq, regardless of the simulated read

depth assessed. Interestingly, higher doses of UV (100 J/m^2) lead to smaller overall coefficients of variation when compared to lower doses of UV (25 J/m^2) (Supplementary Fig. 6). This is likely caused by the greater accumulation of mapped reads within the first 5 kb downstream of the TSS at higher UV doses (Fig. 1e&f) and suggests that BruUV-seq performs better at higher doses of UV.
